# Characterization of a Novel Compound That Stimulates STING-Mediated Innate Immune Activity in an Allele-Specific Manner

**DOI:** 10.3389/fimmu.2020.01430

**Published:** 2020-07-08

**Authors:** Jinu Abraham, Sara Botto, Nobuyo Mizuno, Kara Pryke, Bryan Gall, Dylan Boehm, Tina M. Sali, Haihong Jin, Aaron Nilsen, Michael Gough, Jason Baird, Marita Chakhtoura, Caroline Subra, Lydie Trautmann, Elias K. Haddad, Victor R. DeFilippis

**Affiliations:** ^1^Vaccine and Gene Therapy Institute, Oregon Health and Science University, Portland, OR, United States; ^2^Veterans Affairs Medical Center, Portland, OR, United States; ^3^Integrated Therapies Laboratory, Earle A. Chiles Research Institute, Portland, OR, United States; ^4^Department of Medicine, Drexel University College of Medicine, Philadelphia, PA, United States; ^5^U.S. Military HIV Research Program, Walter Reed Army Institute of Research, Silver Spring, MD, United States; ^6^Henry M. Jackson Foundation for the Advancement of Military Medicine, Bethesda, MD, United States

**Keywords:** interferon, cytokine, cytosolic DNA sensing, STING, adjuvant

## Abstract

The innate immune response to cytosolic DNA involves transcriptional activation of type I interferons (IFN-I) and proinflammatory cytokines. This represents the culmination of intracellular signaling pathways that are initiated by pattern recognition receptors that engage DNA and require the adaptor protein Stimulator of Interferon Genes (STING). These responses lead to the generation of cellular and tissue states that impair microbial replication and facilitate the establishment of long-lived, antigen-specific adaptive immunity. Ultimately this can lead to immune-mediated protection from infection but also to the cytotoxic T cell-mediated clearance of tumor cells. Intriguingly, pharmacologic activation of STING-dependent phenotypes is known to enhance both vaccine-associated immunogenicity and immune-based anti-tumor therapies. Unfortunately, the STING protein exists as multiple variant forms in the human population that exhibit differences in their reactivity to chemical stimuli and in the intensity of molecular signaling they induce. In light of this, STING-targeting drug discovery efforts require an accounting of protein variant-specific activity. Herein we describe a small molecule termed M04 that behaves as a novel agonist of human STING. Importantly, we find that the molecule exhibits a differential ability to activate STING based on the allelic variant examined. Furthermore, while M04 is inactive in mice, expression of human STING in mouse cells rescues reactivity to the compound. Using primary human cells in *ex vivo* assays we were also able to show that M04 is capable of simulating innate responses important for adaptive immune activation such as cytokine secretion, dendritic cell maturation, and T cell cross-priming. Collectively, this work demonstrates the conceivable utility of a novel agonist of human STING both as a research tool for exploring STING biology and as an immune potentiating molecule.

## Introduction

The innate immune response is a rapid cell-based reaction to microbial infection and diseased cellular states that predominantly involves secretion of immunologically functional cytokines. This results from activation of transcription factors or proteolytic caspases at the terminus of intracellular signaling cascades. These signaling pathways are initiated by pattern recognition receptor (PRR) proteins that directly engage and are induced by pathogen- or damage-associated molecular patterns (PAMPs, DAMPs). Among the most potent cytokines are the type I interferons (IFN-I) including IFNβ and multiple IFNα subtypes. IFN-I bind to the nearly ubiquitous IFNα/β receptor (IFNAR) which then activates via Janus kinases (JAK) the transcription factors signal transducer and activator of transcription 1 and 2 (STAT1/2) and IFN regulatory factor 9. The IFNAR-JAK-STAT pathway leads to the transcription of numerous IFN-stimulated genes (ISGs) that exhibit diverse phenotypic effects including generation of antiviral states and coordinating adaptive immunity (1). IFNs are thus essential for combating infectious (especially viral) diseases, anti-tumor T cell responses, and maintaining tissue homeostasis.

Synthesis of IFNβ mRNA specifically requires the transcription factor IFN regulatory factor 3 (IRF3) (2). Activation of this involves phosphorylation by the multi-target kinase TANK binding kinase 1 (TBK1). This, in turn, is activated by one of three adaptor proteins (TRIF, MAVS, and STING) that serve as signal integrators from upstream PRRs (3). Stimulator of IFN genes (STING, also called MITA, ERIS, MPYS, TMEM173) is an ER-associated protein that functions as an adaptor for signals from PRRs that react to cytosolic dsDNA [reviewed in (4)]. Importantly, STING is itself a PRR engaged by cyclic dinucleotides (CDNs) that are synthesized both during bacterial infection as well as by cyclic GMP-AMP synthase (cGAS), a cellular nucleotidyl transferase that is activated after binding to cytosolic DNA (5–7). cGAS uses ATP and GTP to produce a cyclic GMP-AMP molecule that contains G(2',5')pA and A(3',5')pG phosphodiester linkages that engages the C-terminal ligand binding domain (LBD) of dimerized STING proteins. Upon ligand binding, STING recruits TBK1 which phosphorylates STING, TBK1, and IRF3. Activated IRF3 then translocates to the nucleus where it drives synthesis of mRNA for IFNβ and a subset of ISGs, often in concert with other transcription factors such as NF-κB (8) and STAT6 (9) that potentiate expression of additional proinflammatory genes.

The STING-dependent activation of type I IFNs as well as other pro-inflammatory cytokines generates cellular and tissue states that are adverse for virus replication (10). Additionally, transient and localized activation of STING can also lead to stimulation of antigen presenting cell (APC) phenotypes involving cytokine, effector, and costimulatory protein expression that promote antigen uptake and processing and lymph node trafficking, and ultimately facilitate establishment of adaptive immune responses. As such, STING-dependent processes are important for antibody and cytotoxic T cell-mediated activity against infecting microbes as well as tumor cells [reviewed in (11)]. Intriguingly, STING activation can be triggered pharmacologically by synthetic small molecules and engineered macromolecules (12). This represents a potentially formidable strategy for eliciting broad-spectrum antiviral activity (13), generating anti-tumor immunity (14), and enhancing vaccine immunogenicity (15). In light of this, numerous efforts are underway to discover novel and safe STING-based immunomodulators that can be utilized for potentiating desirable clinical outcomes. Unfortunately, significant STING polymorphism exists in the human population, which affects both the molecular responses induced by the protein's activation and the degree of sensitivity to stimulatory ligands (16). Consequently, this can greatly impact the efficacy and safety of molecular entities pursued for clinical purposes. Here we describe a novel small molecule that activates the IFN-I response by way of STING that is differentially active in naturally occurring variants of the protein. In primary human cells this compound is also capable of inducing innate activity that is consistent with facilitation of adaptive immunity and as such may represent a new STING-directed molecular entity with clinical applications.

## Results

### M04 Is a Small Molecule That Activates Type I IFN Signaling in Human Cells

**Figure 1:**
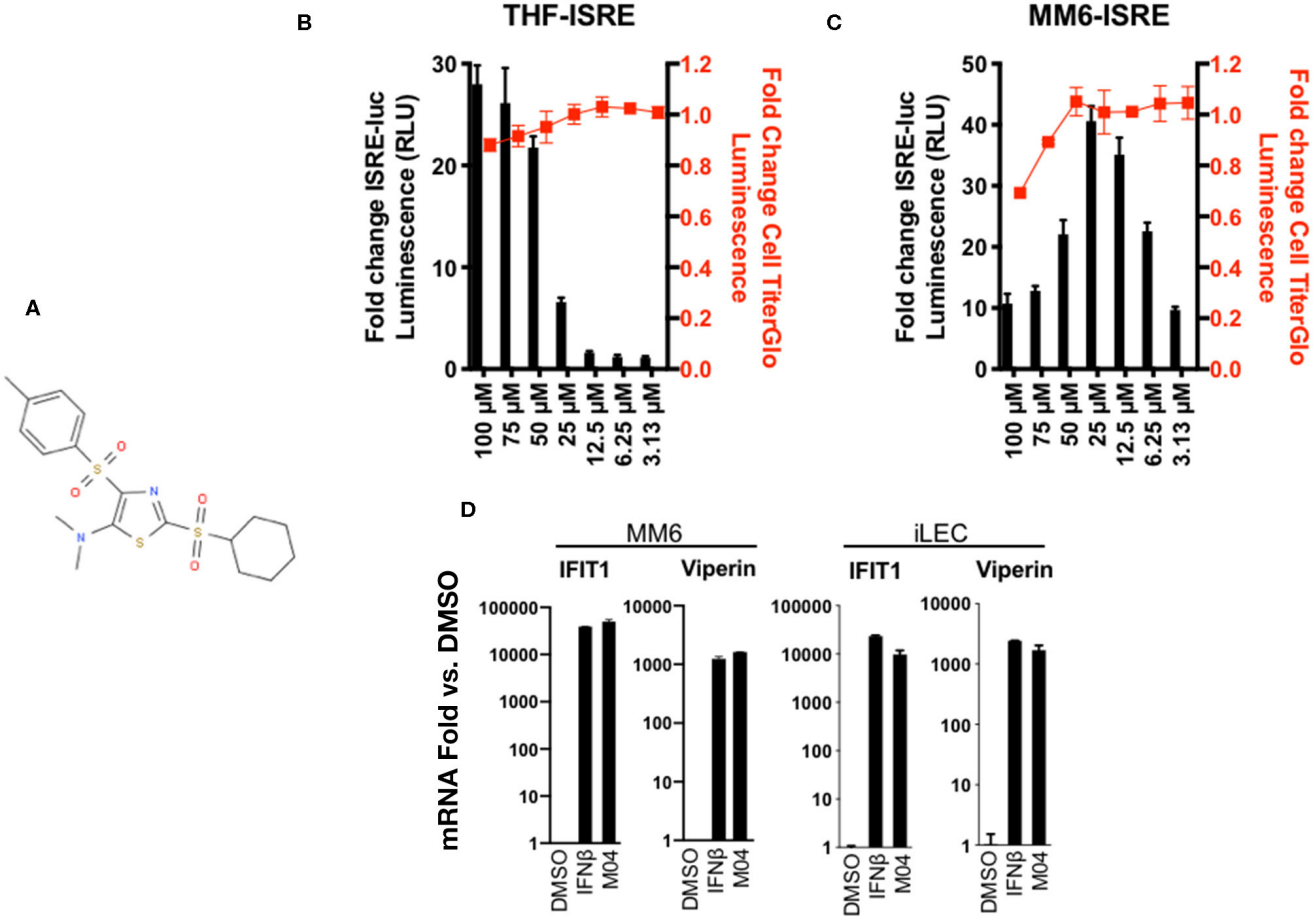
Dose-Dependent Activation of Type I Interferon-Mediated Signaling and Cytotoxicity of M04 in Human Cells. **(A)** Chemical structure of 2-(cyclohexylsulfonyi)-N,N-dimethyl-4-tosylthiazol-5-amine (”M04"); **(B)** ISRE-dependent expression of Luciferase (LUC) as well as relative cellular viability as determined by Cell Titer Glo in THF **(B)** and MM6 **(C)** cells exposed to M04 at indicated concentrations (μM) for 8 h (RLU) or 24 h (Cell Titer Glow). Values presented are mean fold change ± SD relative to cells treated with 1% DMSO (RLU; black bars; left y-axis). Cell viability data are expressed as relative signal detected in DMSO-treated cells (red squares; right axis). Values displayed are based on four replicates; **(D)** Fold changes of IFIT1 or Viperin mRNA relative to 1% DMSO treatment in immortalized lymphatic endothelial cells (iLEC) or MM6 following 8 h exposure to 1000U/mL IFNβ or 50 μM M04 as indicated. Presented values represent average ± SD mRNA fold changes relative to cells exposed to untreated cells from duplicate experiments.

Previous work from our group described a high throughput screen (HTS) undertaken to identify novel small molecules capable of stimulating the type I IFN response in human cells (17–19). From a library of >51,000 compounds, the second most reactive hit was 2-(cyclohexylsulfonyl)-N,N-dimethyl-4-tosylthiazol-5-amine (we term this M04 for simplicity; Figure 1A), which has a MW of 428.6 and LogP of 4.17. The original screen was performed on telomerase-transduced human foreskin fibroblasts (THF) into which a reporter cassette encoding the firefly luciferase (LUC) open reading frame controlled by IFN-stimulated responsive elements (ISRE) was also stably introduced. To validate the HTS results we therefore measured LUC expression in these cells following exposure to a range of M04 doses and, in parallel, cytotoxicity. As shown in Figure 1B, LUC signal was maximal at 100 μM with only minimal loss in cell viability. To examine efficacy of the molecule on human cells of a distinct ontology we employed myeloid-derived MonoMac6 (MM6) cells (20). These were stably transduced with the same ISRE-LUC reporter cassette and treated with a similar range of M04 doses. As shown in Figure 1C, MM6-ISRE exhibited higher sensitivity to M04 with maximum signal observed at 25 μM. While no detectable toxicity was observed at that concentration, higher doses showed significant cell death.

While these data show that M04 can clearly activate expression of an artificial IFN-sensitive reporter, we next aimed to establish whether the molecule is also capable of inducing transcription of endogenous IFN-stimulated genes (ISGs). For this we exposed MM6 cells to M04 as well as IFNβ and used semi-quantitative reverse transcriptase PCR (qPCR) to measure induced levels of IFIT1 (21) and Viperin (RSAD2) (22). As shown in Figure 1D, M04 led to levels of IFIT1 and Viperin mRNA synthesis that resembled those induced by IFNβ. Since results thus far indicated that M04 was able to trigger IFN-associated activity in stromal and myeloid-derived cells, we next examined whether an unrelated cell type was also responsive to the molecule. For this we performed qPCR using immortalized human lymphatic endothelial cells (iLEC) treated as described for MM6 and observed similar levels of ISG induction (Figure 1D). Taken together, these data indicate that M04 is a novel small molecule capable of stimulating IFN-dependent responses across human cell types in a dose dependent manner without significant cytotoxicity at its active concentrations.

### M04-Mediated Innate Stimulation Requires Activation of TBK1 and IRF3

**Figure 2:**
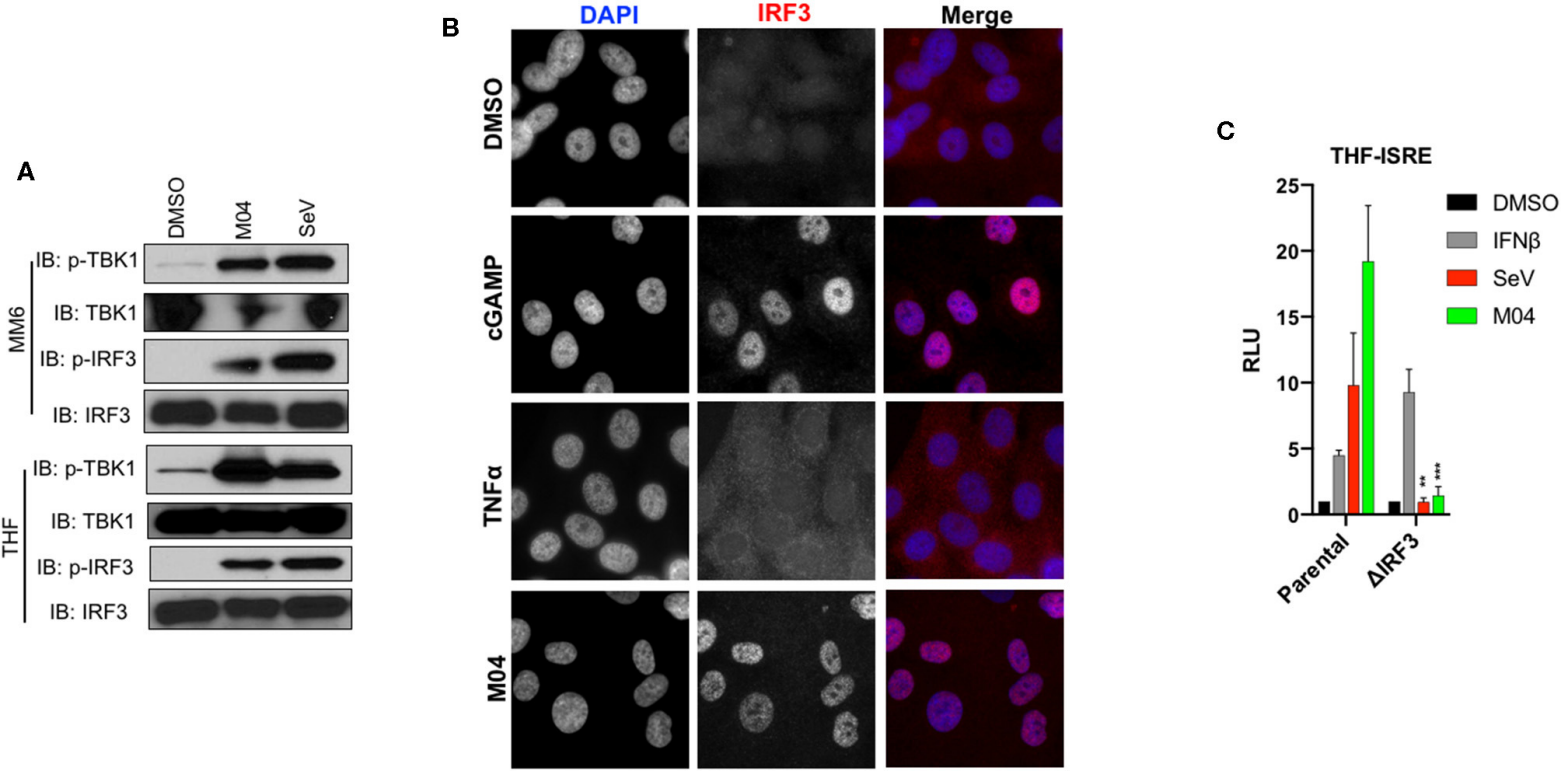
M04 induces canonical activation of IRF3 which is essential to reporter signal generated by the compound. **(A)** Immunoblot showing phosphorylation status of TBK1 Ser172 and IRF3 Ser386 as well as corresponding total protein levels in MM6 cells (left) and THF (right) exposed for 4 h to 1% DMSO, 50 μM M04, or 1,000 HAU/mL SeV as indicated; **(B)** Indirect immunofluorescence showing subcellular localization of IRF3 in THF exposed for 4 h to 1% DMSO, transfected 2'3'cGAMP (10 μg/mL), 100 ng/mL TNFα, or 50 μM M04; **(C)** Reporter assay illustrating IFN-dependent LUC induction following overnight treatment with 1% DMSO, 1,000 U/mL IFNβ, 1,000 HAU/mL SeV, or 50 μM M04 in parental cells as well as those from which IRF3 was deleted as indicated. Data presented are mean ± SD relative luminescence units (RLU) using signal from DMSO-treated cells based on quadruplicate measurements. Student's *T*-test was used to compare RLU in the parental and ΔIRF3 cells **P < 0.01; ***P < 0.001.

Conventional initiation of the type I IFN response involves activation of the IRF3 transcription factor via phosphorylation of serine residues by TBK1 which then enables nuclear translocation and transcription of IFNβ (23). To examine whether this activity occurs following treatment with M04 we performed immunoblotting (IB) using whole cell lysates from MM6 and THF cells treated with M04 or the RIG-I/MAVS/IRF3-activating stimulus Sendai virus (SeV) (24). As shown in Figure 2A, both stimuli led to the phosphorylation of TBK1 and IRF3, indicating inducible activation of both proteins. Since activated IRF3 must accumulate in the nucleus to drive IFN and ISG transcription, we next examined its subcellular localization using indirect immunofluorescence assay (IFA). As shown in Figure 2B, THF cells treated with M04 as well as the STING/IRF3 agonist 2'3' cyclic GMP-AMP (cGAMP), but not the cytokine TNFα, displayed obvious nuclear IRF3 protein, consistent with its typical activated status.

While these data demonstrate standard activation of the TBK1-IRF3 signaling axis, whether this is essential to the IFN-associated innate induction triggered by M04 cannot be formally concluded. To address this, we utilized previously published THF reporter cells from which the IRF3 protein was deleted using CRISPR/Cas9-mediated genome editing (19). As shown in Figure 2C, derivative mutant cells are capable of producing reporter signal following treatment with IFNβ, which indicates that JAK/STAT signaling is intact. However, neither SeV nor M04 were able to elicit measurable reporter expression in these cells indicating that IRF3 is required for the induction of IFN-dependent signaling by both stimuli. Based on these data we conclude that M04 stimulates type I IFN responses through the canonical and necessary activation of TBK1 and IRF3.

### M04 Does Not Stimulate Activation of Canonical NF-κB-Associated Transcription

**Figure 3:**
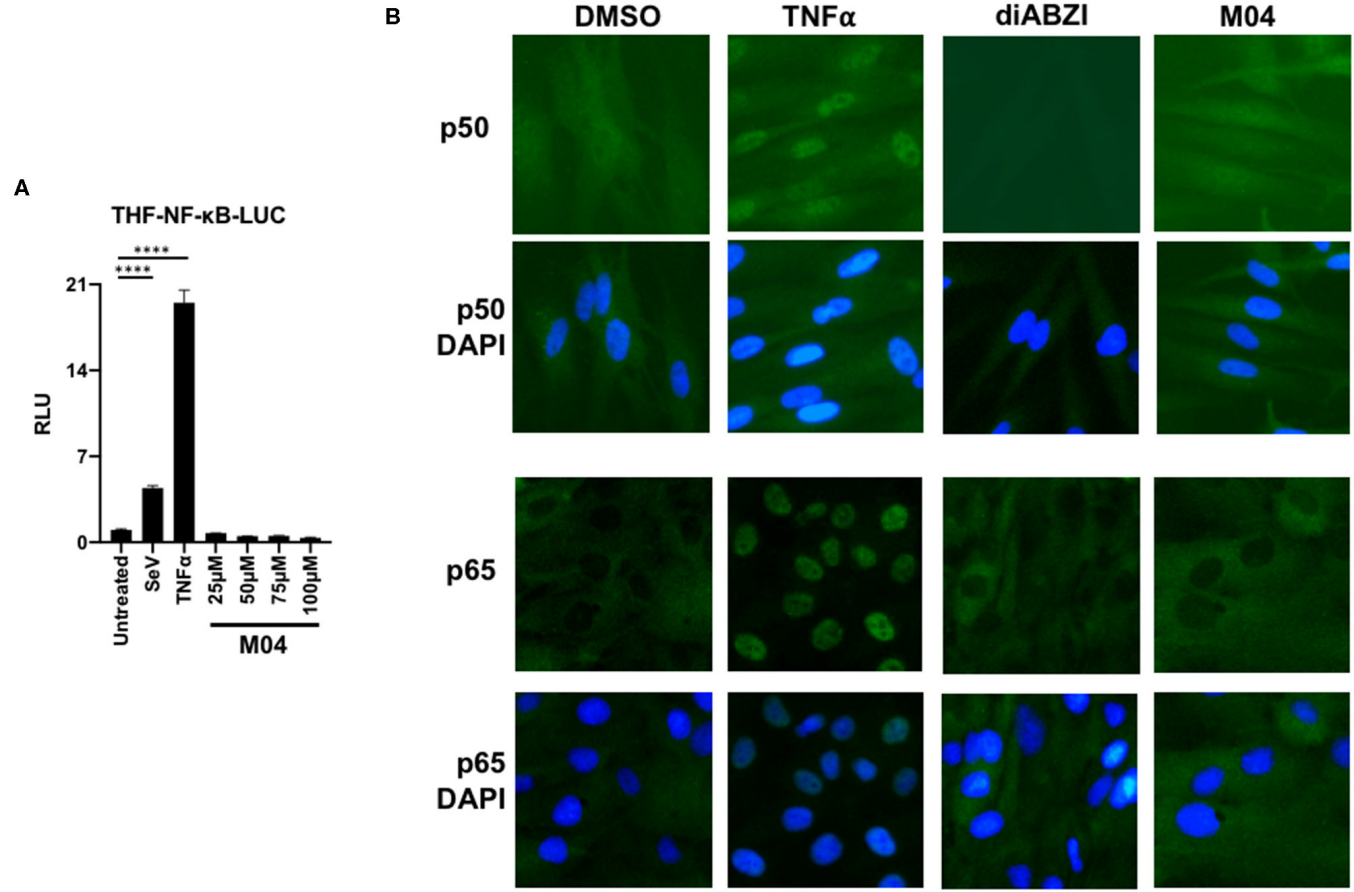
M04 does not Activate NF-κB-Dependent Processes. **(A)** Reporter assay using THF cells responsive to activated NF-κB showing induction of LUC expression following 8 h treatment with 160 HAU/mL SeV, 10 ng/mL TNFα, or the indicated concentration of M04. Values displayed are average fold changes (±SD) based on four replicates compared to DMSO-treated cells; **(B)** Indirect immunofluorescence showing subcellular localization of NF-KB P65 subunit in THF exposed for 4 h to DMSO, 100 ng/mL TNFα, or 75 μM M04. Statistical significance between treated and untreated cells was then calculated using Student's *T*-test ****p < 0.0001.

The transcription factor NF-κB is activated by signaling initiated from multiple PRRs (including many that are also IRF3-directed) (25). Importantly, the protein also contributes to the expression of numerous proinflammatory cytokines, including type I IFNs (8, 9). Since M04 leads to conventional activation of IRF3, we therefore asked whether it also stimulates NF-κB. To address this we first exposed M04 to THF stably transduced with an NF-κB-dependent LUC reporter as described (18). As shown in Figure 3A, the compound was unable to activate LUC expression in these cells at a range of doses, in contrast to stimuli known to induce NF-κB such as SeV or the cytokine TNFα. Next, we examined whether M04 could induce nuclear accumulation of the NF-κB subunit proteins P50 and P65, a hallmark of canonical activation. For this we exposed THF to DMSO vehicle, TNFα, the STING ligand di-amidobenzimidazole (diABZI) (26), or M04 and used IFA to visualize subcellular localization of the proteins. As shown in Figure 3B, TNFα, but neither diABZI nor M04 led to nuclear localization of P65 and P50. Collectively, these data indicate that M04 does not lead to activation of NF-κB.

### M04 Activates IRF3 and IFN-Terminal Signaling That Requires STING but Not MAVS, TRIF, or dsDNA PRRs

**Figure 4:**
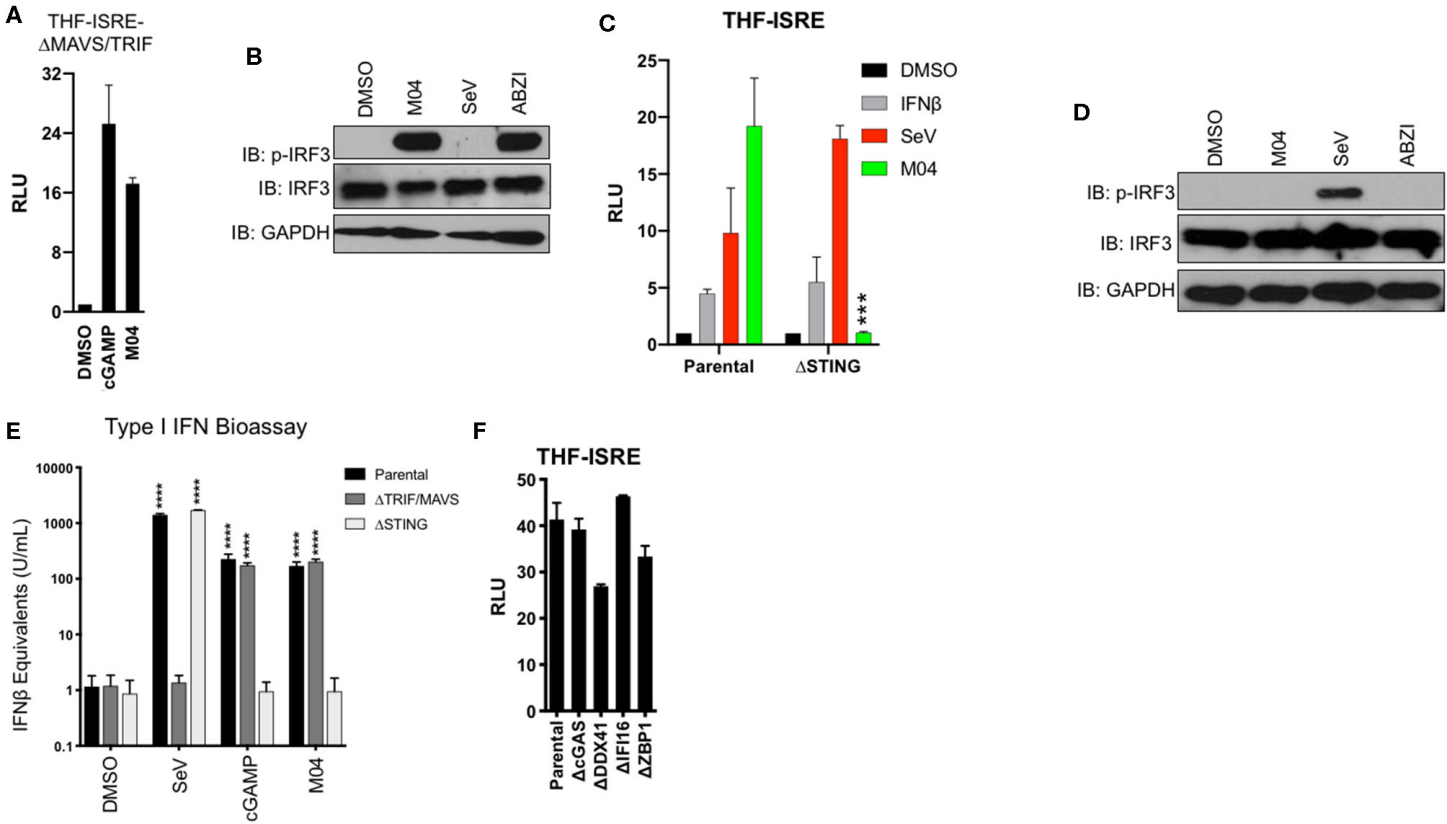
Innate Activation by M04 requires STING but not MAVS, TRIF, or cytosolic DNA PRRs. **(A)** Reporter assay illustrating IFN-dependent LUC induction in THF-ISRE-ΔMAVS/TRIF following overnight treatment with 1% DMSO, transfected cGAMP (10 μg/mL), or 75 μM M04. Data presented are mean ± SD relative luminescence units (RLU) using signal from DMSO-treated cells as the basis (*n* = 4 treatments); **(B)** Immunoblot showing phosphorylation status of IRF3 Ser386, total IRF3, and GAPDH in THF-ISRE-ΔMAVS/TRIF following 8 h treatment with 1% DMSO, 75 μM M04, 1,000 HAU/mL SeV or 25 μM ABZI as indicated; **(C)** Reporter assay illustrating IFN-dependent LUC induction in THF-ISRE-ΔSTING following overnight treatment with 1% DMSO, 1,000 U/mL IFNβ, 1,000 HAU/mL SeV, or 75 μM M04. Data presented are mean RLU ± SD as described above; Student's *T*-test was used to compare RLU ***p < 0.001; **(D)** Immunoblot showing phosphorylation status of IRF3 Ser386, total IRF3 in THF-ISRE-ΔSTING following 4 h treatment with 1% DMSO, 50 μM M04, 1,000 HAU/mL SeV or 25 μM ABZI as indicated; **(E)** Secretion of bioactive type I IFN from parental THF as well as THF-ISRE-ΔMAVS/TRIF and THF-ISRE-ΔSTING treated in triplicate overnight with 1% DMSO, 1,000 HAU/mL SeV, transfected cGAMP (10 μg/mL), or 75 μM M04. Data are expressed as mean concentrations ± SD for IFNβ equivalent units. Statistical significance between treated and untreated cells of similar genetic background was calculated using Student's *T*-test. ****p < 0.0001; **(F)** Reporter assay from WT parental THF-ISRE cells as well as from cells from which indicated dsDNA-specific PRRs were deleted. Values presented are mean fold changes ± SD for duplicates relative to the value for DMSO-treated cells.

Three separate signaling cascades are known to elicit TBK1-IRF3 activation and these are defined by the adaptor proteins MAVS, TRIF, and STING [see (3)]. We therefore explored which, if any, of these proteins are required for M04-mediated induction of IRF3 and IFN. We began by utilizing THF-ISRE cell lines constructed previously that lack both MAVS and TRIF (17). Figure 4A shows that cGAMP [a STING-inducing IRF3/IFN activator (6, 7, 27, 28)] and M04 are able to elicit LUC expression in these cells suggesting that neither TRIF nor MAVS is required for their activity. We next examined whether M04 could activate IRF3 phosphorylation or ISG expression in these cells. In this case we included SeV as a control stimulus to demonstrate knockout as well as linked amidobenzimidazole (ABZI) as a control small molecule STING activator (26). As shown in Figure 4B, M04 and ABZI, but not SeV, were able to induce IRF3 phosphorylation. These results strongly suggest that both MAVS and TRIF are dispensable for M04-mediated IFN induction but that STING may play an important role. To address this issue we employed THF-ISRE from which STING was deleted (17–19). While the reporter cassette in these cells was reactive to IFNβ and SeV as expected, it was not induced by M04 (Figure 4C). Moreover, while SeV led to phosphorylation of IRF3 in these cells, neither M04 nor ABZI elicited a similar response (Figure 4D). To examine this further we assessed type I IFN secretion using a reporter cell-based bioassay on media from treated parental THF as well as those lacking MAVS and TRIF or STING as described (17). We exposed the cells to DMSO, SeV, transfected cGAMP, or M04 and measured secretion of all bioactive type I IFNs using a reporter-based assay. As expected, parental cells secreted IFN-I in response to the three innate stimuli (Figure 4E). Furthermore, MAVS/TRIF-deficient cells did not respond to SeV but were reactive to cGAMP and M04 while STING-deficient cells produced IFN-I in response to SeV but not cGAMP or M04. To explore the innate induction ability of M04 relative to other IRF3-terminal stimuli, we also performed a dose response on THF-ISRE that included SeV (MAVS agonist) and human cytomegalovirus (HCMV; STING agonist). As shown in Supplemental Figure 1, maximum activation by M04 approximates that induced by HCMV at an MOI of 0.25 and is higher than that induced by SeV at up to 160 HA units/mL.

These results indicate that STING, but not MAVS or TRIF is required for M04-mediated innate activation. STING is fundamentally involved in the innate intracellular response to cytosolic dsDNA (10, 29–31). In addition, multiple dsDNA-reactive PRRs including cGAS (32), DDX41 (33), IFI16 (34), and DAI/ZBP1 (35) are known to be associated with or upstream of STING-dependent responses. We therefore next asked whether M04-induced activity required any of these proteins. For this, RLU was measured using previously described THF-ISRE cells lacking each individual PRR after exposure to M04 (17). As shown in Figure 4F, M04 was active on all these cell lines, indicating that none of the deficient proteins are singularly essential for the compound's effects. Based on these results we conclude that M04 activates an IRF3- and IFN-terminal innate immune response in a manner that requires STING but not MAVS, TRIF, or canonical dsDNA PRRs.

### M04 Induces Phosphorylation and ER-Golgi Trafficking of STING

**Figure 5:**
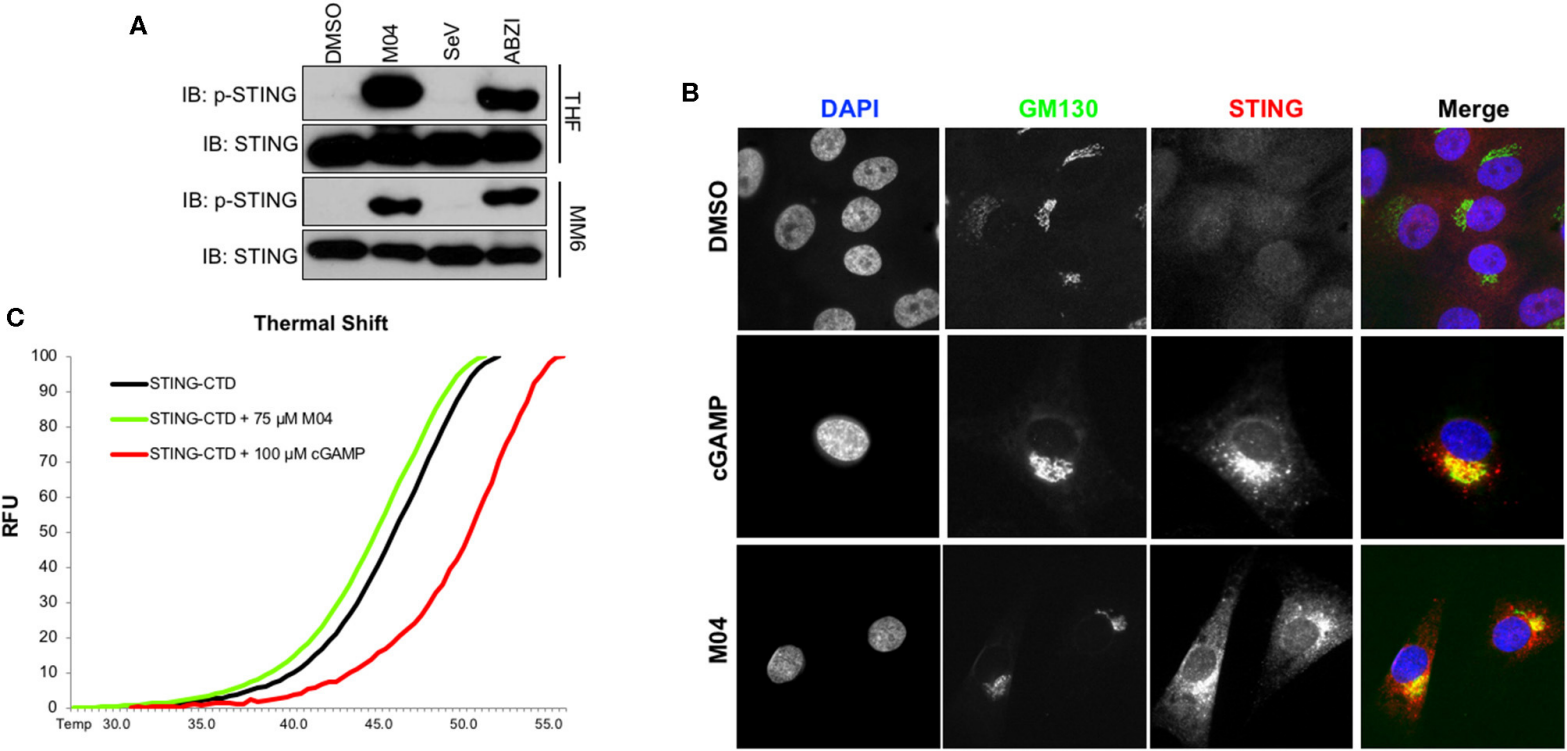
M04 Induces Canonical STING Activation. **(A)** lmmunoblot showing phosphorylation status of STING Ser366 as well as total STING in THF and MM6 following 4 h treatment with 1% DMSO, 50μM M04, 1,000 HAU/mL SeV or 25 μM ABZI as indicated; **(B)** Indirect immunofluorescence showing subcellular localization of Golgi marker GM130 and STING in THF exposed for 4 h to DMSO, transfected cGAMP (10 μg/mL), 100 ng/mL TNFα, or 50 μM M04; **(C)** Melting temperature shifts for human STING-CTD in the presence of DMSO, 75 μM M04, or 100 μM 2'3' cGAMP. Data presented are SYPRO orange relative fluorescent units (RFU).

Canonical activation of STING involves phosphorylation of serine residue 366 (36) followed by translocation from the ER to the Golgi apparatus (37). Since M04 requires STING for IRF3 activation we predicted that the compound leads to these two outcomes. As shown in Figure 5A, immunoblots on whole cell lysates from MM6 and THF cells treated with M04 or ABZI displayed phosphorylation of STING Ser366 whereas lysates from untreated or SeV-treated cells did not. We next performed IFA to examine co-localization of STING with the standard Golgi protein marker GM130. Figure 5B shows that transfection of THFs with cGAMP or treatment with M04 leads to accumulation of STING in regions that also stain positive for GM130. These results are consistent with conventional intracellular activation of STING in response to M04. Since M04 induces IRF3 independently of any examined dsDNA PRRs or cGAMP synthesis, we next explored whether observations could be obtained that indicate direct interaction between the molecule and the STING protein. This was performed as part of previous studies measuring thermal shift of purified STING C-terminal domain (CTD) that includes the ligand binding domain (LBD) (17, 19). We expect that direct contact between the protein and an examined ligand will lead to an increase in the protein's thermal stability that is observable as emission of protein-associated SYPRO Orange at higher temperatures than those in the absence of the ligand (27, 38). As shown in Figure 5C, incubation of purified STING-CTD with cGAMP led to an increase in the temperature at which fluorescence was emitted relative to that with DMSO alone. However, the presence of M04 did not lead to a significant increase in such temperatures relative to that induced by DMSO. These results are not consistent with M04 directly interacting with STING-LBD as does cGAMP. While direct interaction cannot formally be ruled out, determining whether M04 activates STING by engaging the CTD in a manner that does not affect thermal stability or by engaging a region outside the CTD will require different experimental approaches such as affinity tagging and protein pulldown. Unfortunately, the innate activity of M04 is highly sensitive to chemical modification as indicated by the absence of ISRE activity in a group of 13 M04 derivatives as shown in Supplemental Figure 2. As such, adding moieties such as biotin to M04 will likely not represent appropriate pulldown bait.

### M04 Stimulatory Capacity Is Dependent on STING Polymorphic Variant

**Figure 6:**
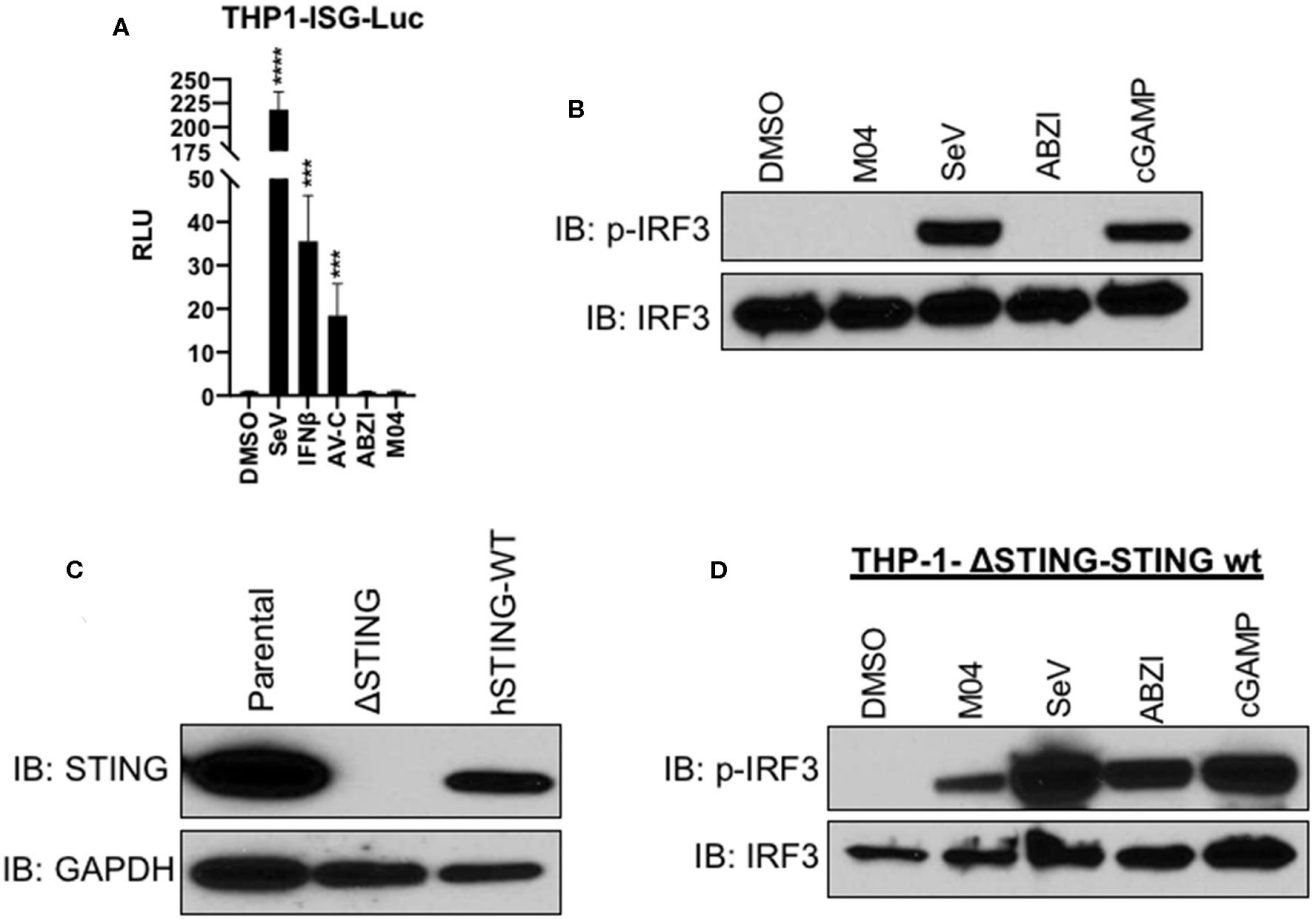
Responsiveness to M04 can be Conferred through Introduction of WT STING Allelic Variant. **(A)** Reporter assay illustrating IFN-dependent LUC induction in THP-1-ISG-Lucia following overnight treatment with 1% DMSO, 1,000 HAU/mL SeV, 1,000 U/mL IFNβ, 75 μM TRIF agonist AV-C, 25 μM ABZI, or 75 μM M04. Data presented are mean ± SD relative luminescence units (RLU) using signal from DMSO-treated cells as the basis (*n* = 4 treatments). Student's *T*-test was used to compare RLU ***p < 0.001, ****p < 0.0001; **(B)** lmmunoblot showing phosphorylation status of IRF3 Ser386 and total IRF3 in THP-1 whole cell lysates following 4 h treatment with 1% DMSO, 50 μM M04, 1,000 HAU/mL SeV, 25 μM ABZI, or 10 μg/mL cGAMP as indicated; **(C)** lmmunoblot showing expression of endogenous or ectopically expressed WT hSTING in THP-1 as indicated. **(D)** Immunoblot showing phosphorylation status of IRF3 Ser386 in THP-1 cells from which endogenous STING was deleted and WT STING stably introduced following indicated treatment as described above.

In human populations multiple amino acid variants of STING are known to exist and these can be mechanistically associated with a range of phenotypic outcomes at the levels of molecular function and disease state [reviewed in (39)]. Since both THF and MM6 cells exhibit the most common and CDN-reactive STING allele (STING-WT), we chose to examine M04 activity in the presence of another variant. For this we used THP-1 promonocytic cells, which possess the R71H-G230A-R293Q (STING-HAQ) allele (7, 16, 40). We first used commercially available, IFN-sensitive THP-1-ISG-Lucia reporter cells. LUC signal was produced by these cells when exposed to SeV, IFNβ, a small molecule agonist of the TRIF pathway termed AV-C (18), but not M04 nor, interestingly, ABZI (Figure 6A). These results suggest that THP-1 cells are not responsive to these two compounds and this was validated by immunoblotting which showed that while SeV treatment led to S386-phosphorylated IRF3, neither M04 or ABZI did (Figure 6B). However, the cells were able to react to cGAMP, indicating that STING signaling is operational in these cells (Figure 6B). Based on this we hypothesized that the endogenous STING-HAQ protein was incapable of reacting to M04 and decided to ask whether ectopic expression of STING-WT could rescue M04 responsiveness in THP-1 cells. To pursue this, we first employed CRISPR/Cas9 to construct a THP-1 line from which the endogenous STING-HAQ protein was deleted and then used a lentivector to stably introduce into these edited cells a constitutively expressed open reading frame encoding the STING-WT protein (Figure 6C). These cells were then treated with SeV, M04, ABZI, or cGAMP and immunoblotting performed to examine IRF3 phosphorylation. As shown in Figure 6D, expression of STING-WT rendered the cells responsive to M04, suggesting that the compound is able to stimulate activity of this, but not the HAQ protein variant.

**Figure 7:**
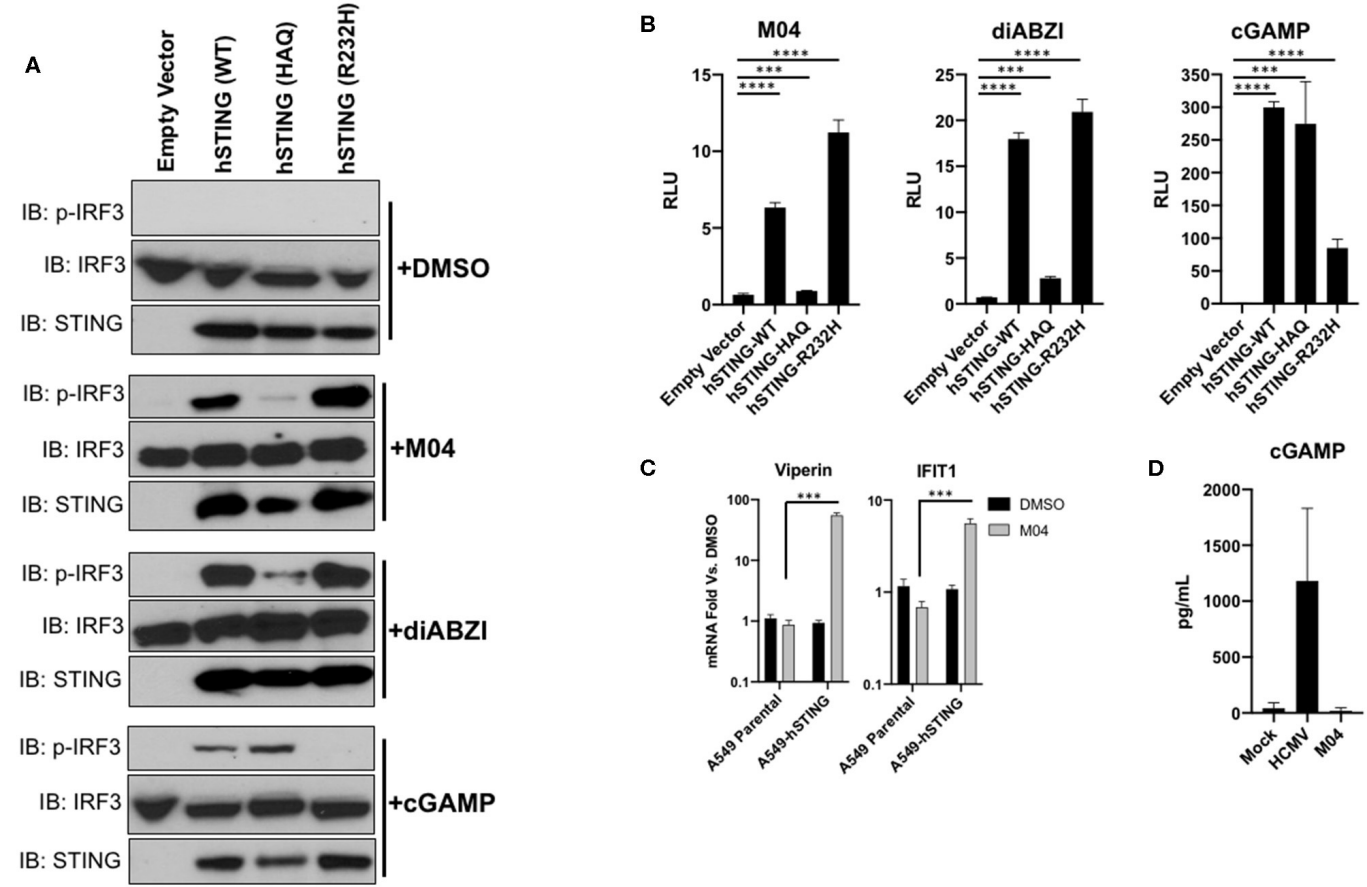
Transient transfection of vectors encoding WT and R232H but not HAQ hSTING confer responsiveness to M04. **(A)** lmmunoblot from HEK293T whole cell lysates showing expression of indicated STING variants following transient transfection, S386 phosphorylation status of IRF3 and total IRF3. Cells were left untreated or exposed to 75 μM M04, 100 nM diABZI, or 10 μg/mL cGAMP as indicated; **(B)** Reporter assay using cells (*n* = 4) treated as described in **(A)**. Values displayed are mean fold changes ± SD relative to cells transfected with empty vector; **(C)** Expression of IFIT1 and Viperin mRNA as determined by qPCR in parental A549 cells as well as those transduced with hSTING following treatment with 1% DMSO or 75 μM M04. Data are mean fodl changes ± SD relative to DMSO-treated cells based on duplicates; **(D)** Synthesis of cGAMP by A549-hSTING cells as determined by ELISA following overnight treatment with 1% DMSO, HCMV, or M04. Data presented are mean pg/mL ± SD based on duplicate samples. Student's *T*-test was used to compare RLU and mRNA levels ***p < 0.001, ****p < 0.0001.

To verify the differential responsiveness of the protein variants to M04 using an independent method, we employed transiently transfected HEK293T cells. These cells are deficient in endogenous STING and as such will only respond to STING inducers during ectopic expression of the protein (29, 31). We constructed plasmid vectors that encode STING-WT, STING-HAQ, or STING-R232H (the third most common STING variant). We then transfected these along with a IFN-responsive LUC reporter vector and exposed the cells to DMSO, M04, cGAMP, or diABZI, a derivative of ABZI shown to be reactive with STING-HAQ (26). We then examined IRF3 phosphorylation and measured LUC expression from whole cell lysates. As shown in Figure 7A, while transfection of the vectors alone did not activate IRF3 phosphorylation, all three stimuli led to phosphorylation of IRF3 in the presence of STING-WT. However, M04 and diABZI elicited only weakly detectable phosphorylation in the presence of STING-HAQ. Furthermore, cGAMP appeared to induce a strong response in the presence of STING-HAQ and STING-WT but not STING-R232H (Figure 7B). This was surprising but consistent with results showing that this allele is comparatively less responsive to cGAMP (7, 16). These results were reflected in the IFN-dependent reporter signal with M04 and diABZI generating detectable signal in the presence of STING-WT and STING-R232H but weak or no signal in STING-HAQ transfected cells and cGAMP inducing highest LUC signal in STING-WT and STING-HAQ. These results also align with those generated in THP-1 cells.

A549 lung epithelial cells suppress expression of the endogenous STING mRNA (41) and, consequently, do not respond to M04 (Figure 7C). We therefore asked whether stable introduction of STING into these cells using methods described above could also rescue M04 responsiveness. As shown in Figure 7C, stable expression of hSTING-WT restores M04-associated ISG expression. Results described so far including these suggest that M04 activates STING in a manner that is independent of DNA PRRs (Figure 4F) and binding to the protein's CTD (Figure 5C). To rule out the unlikely possibility that M04 stimulates cGAS-independent synthesis of cGAMP we treated A549 cells with M04 and harvested lysates to measure cGAMP by ELISA. Figure 7D shows that while infection with HCMV induces cGAMP synthesis as described (42), treatment with M04 does not. Collectively, our results indicate that M04 activates STING in a cGAMP-independent manner either by directly engaging the protein or by stimulating a cellular factor common to THP-1, HEK293T, and A549 cells that regulates STING function.

### hSTING Confers Responsiveness to M04 Across Species

**Figure 8:**
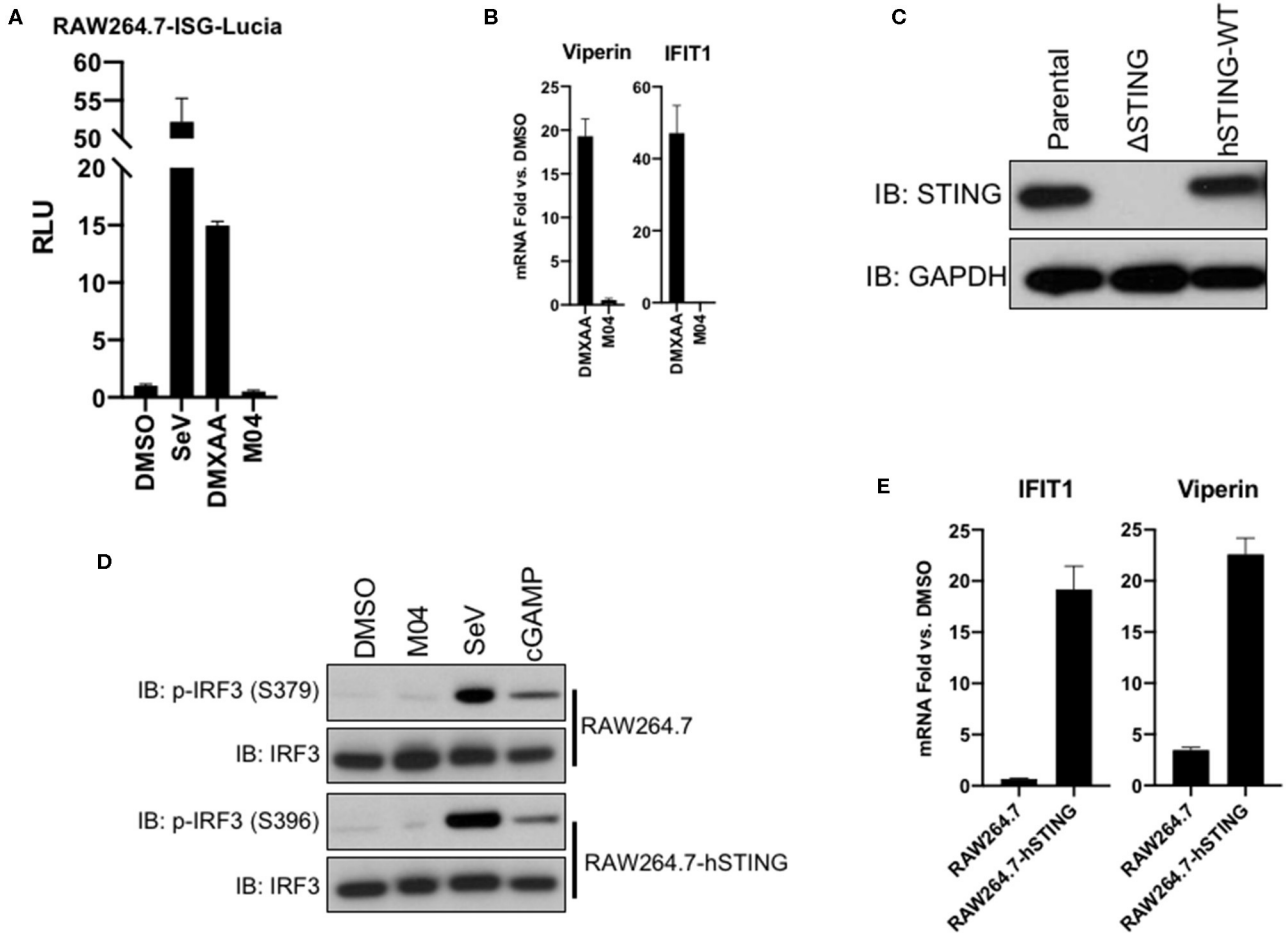
Responsiveness to M04 can be conferred to murine cells by ectopic expression of human STING-WT variant. **(A)** Reporter assay illustrating IFN-dependent LUC induction in RAW264.7-ISG-Lucia cells followni g overnight treatment with 1% DMSO, 160 HAU/mL SeV, 25 μM DMXAA, or 75 μM M04.Data presented are mean ± SD relative luminescence units (RLU) using signal from DMSO-treated cells as the basis (*n* = 4 treatments); **(B)** qPCR examining *in vivo* ISG induction following IP injection of DMXAA or M04; **(C)** lmmunoblot showing expression of endogenous or ectopically expressed hSTING-WT in RAW264.7 cells as indicated; **(D)** lmmunoblot showing phosphorylation status of IRF3 Ser379 and Ser396 as well as total IRF3 in RAW264.7-hSTING cells following 4 h treatment with 1% DMSO, 75 μM M04, 160 HAU/mL SeV, or transfection of cGAMP as indicated; **(E)** qPCR examining transcription of IFIT1 or Viperin following overnight treatment of parental RAW264.7 and RAW264.7-hSTING cells with 75 μM M04 (*n* = 3). Data presented are mean fold changes ± SD of mRNA relative to cells treated with 1% DMSO.

Given that the efficacy of M04 associates with amino acid polymorphisms in the human STING allele, we believed it unlikely that the compound triggers an innate response in mouse cells. To address this, we first examined a commercially available RAW264.7 murine macrophage-like line that expresses an IFN-dependent reporter (RAW264.7-ISG-Lucia). In these, SeV and DMXAA [a mouse-specific STING agonist (43)], but not M04 were able to induce reporter expression (Figure 8A). To examine whether the compound might still be active in an *in vivo* setting, we injected it intraperitoneally into C57BL/6 mice and harvested spleens after 5 h. While DMXAA was able to induce expression of Viperin and IFIT1 relative to DMSO-vehicle treated control mice, we observed no upregulation of these genes in response to M04 (Figure 8B). Since previous work has demonstrated functionality of hSTING in mouse cells (44), we next utilized a delete-and-replace approach as described above to see if responsiveness to M04 could be conferred to RAW264.7 cells by ectopic expression of hSTING-WT. Figure 8C shows expression of endogenous or human STING-WT in parental RAW264.7 cells as well as following CRISPR-mediated knockout and target hSTING protein stable introduction by lentivector. These cells were then exposed to DMSO, M04, SeV, or cGAMP. As shown in Figure 8D, SeV and cGAMP led to similar levels of phosphorylation IRF3 on serine residues 379 and 396. Surprisingly, however, M04 did not elicit detectable phosphorylation of IRF3 in either cell type. Since it is possible that IRF3 is activated by phosphorylation of C-terminal serine residues not detectable by available antibodies, we also examined M04-mediated induction of ISGs in these cells. As shown in Figure 8E, the compound induced minimal or no ISG expression in parental cells but substantial amounts in cells expressing hSTING. From these data we conclude that M04 leads to hSTING effects that can activate innate responses in non-human cells.

### M04 Is Able to Elicit Secretion of Pro-inflammatory Cytokines From Primary Human Cells

**Figure 9:**
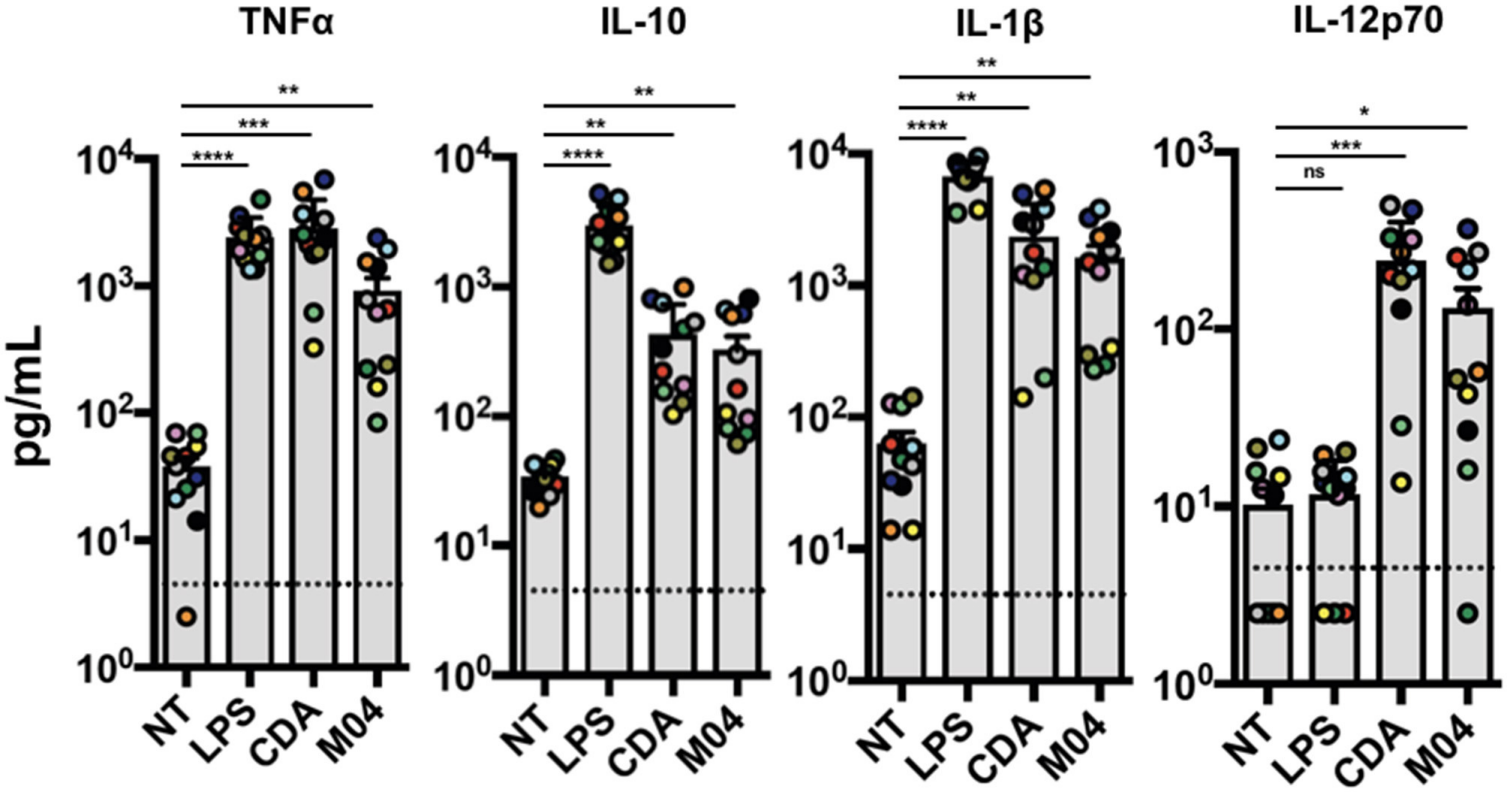
Induction of cytokine expression by M04 on human primary cells. Peripheral blood mononuclear cells were harvested from ten human donors and treated overnight with 1OO ng/mL LPS, 10 μg/mL cyclic di-AMP (CDA), or 75 μM M04 as indicated. Luminex multiplex assay was then used to measure levels of TNFa, IL-10, IL-1β, or IL12p70 in cell culture supernatant. Donor specific data are indicated by colored circles. Statistical significance between treated and untreated cells was then calculated using Student's *T*-test. *p < 0.5, **p < 0.01, ***p < 0.001; ****p < 0.0001.

STING agonism represents a potentially impactful pharmacologic strategy in the context of facilitating adaptive immune responses. However, thus far we only describe induction of STING-dependent responses in immortalized or telomerized cell lines. We therefore wished to determine whether M04 could activate innate phenotypes relevant for clinical uses. However, since M04 is inactive in conventional murine models, tractable options for exploring *in vivo* effects are not available. In light of this, we chose to utilize human peripheral blood mononuclear cells (PBMCs) to explore M04-mediated cellular outcomes. Since STING agonists are being pursued clinically as anti-cancer immunotherapeutics (45), we evaluated the response of a relevant population of patients with locally advanced or borderline resectable pancreatic cancer (46). PBMC isolated from patients prior to treatment were exposed to M04 overnight and media harvested for a multiplex assay to measure cyto/chemokines secreted in response to treatment. Cells were also left untreated, exposed to cyclic di-AMP (CDA) as a STING-specific positive control, or LPS as a STING-independent, IRF3-stimulating control. As shown in Figure 9, M04 significantly induced secretion of TNFα, IL-1β, IL12p70, and IL-10. Moreover, the patterns of M04-associated induction more closely resembled those observed for CDA than LPS, consistent with STING dependence of the two stimuli. Based on this we conclude that M04 is capable of inducing innate responses in primary human cells.

### M04 Triggers Expression of Human Dendritic Cell Maturation Markers

**Figure 10:**
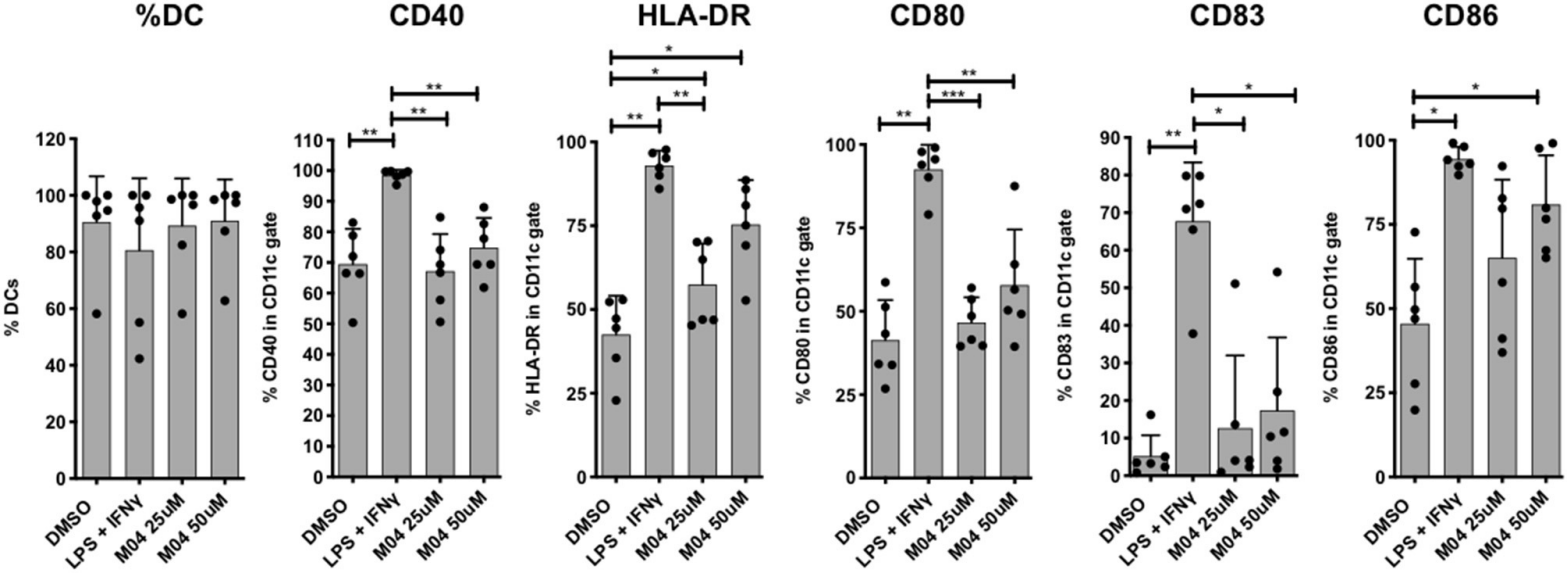
M04 induces HLA and costimulatory molecule upregulation on human monocyte-derived dendritic cells. Human monocyte-derived dendritic cells (DCs) differentiated from healthy human PBMCs were treated with 1% OMSO or stimulated with 0.5 μg/ml LPS plus 40 ng/ml IFN-y and 25 μM or 50 μM M04 for 24 h. DCs were harvested (% DCs indicated PBMCs indicated at left) and analyzed by flow cytometry for the upregulation of surface C040, HLA-DR, CD80, CD83, and CD86 as indicated. Values are presented as mean ± the standard deviations (mean ± SD) for the indicated marker from 6 individual donors across 3 independent experiments (donor-specific values are represented by closed circles). *p < 0.5, **p < 0.01, ***p < 0.001.

Dendritic cells (DC) are essential for the establishment of adaptive immunity based on their capacity to present antigens and secrete immunologically potent cytokines. This process first involves their maturation, as denoted by surface marker expression, in response to appropriate innate immune stimuli that are often indicative of microbial infection or diseased cells. We therefore asked whether M04 was capable of eliciting induction of maturation markers on human cells. For this we employed PBMCs from six healthy human donors in an ex vivo culture system. Immature monocyte-derived DCs cultured in IL-4 and GM-CSF were treated with two doses of M04. Control stimuli included DMSO (negative) and LPS + IFNγ (positive). Flow cytometry was then used to quantify expression of CD40, HLA-DR, CD80, CD83, and CD86. As shown in Figure 10, expression of HLA-DR and CD86 were significantly elevated after M04 exposure relative to vehicle-treated cells. Surprisingly, however, the compound did not similarly induce CD40, CD80, or CD83. These results suggest that M04 behaves as an innate stimulus that is capable of facilitating maturation of APCs and may thus exhibit adjuvant properties.

### M04 Enables T Cell Cross Priming

**Figure 11:**
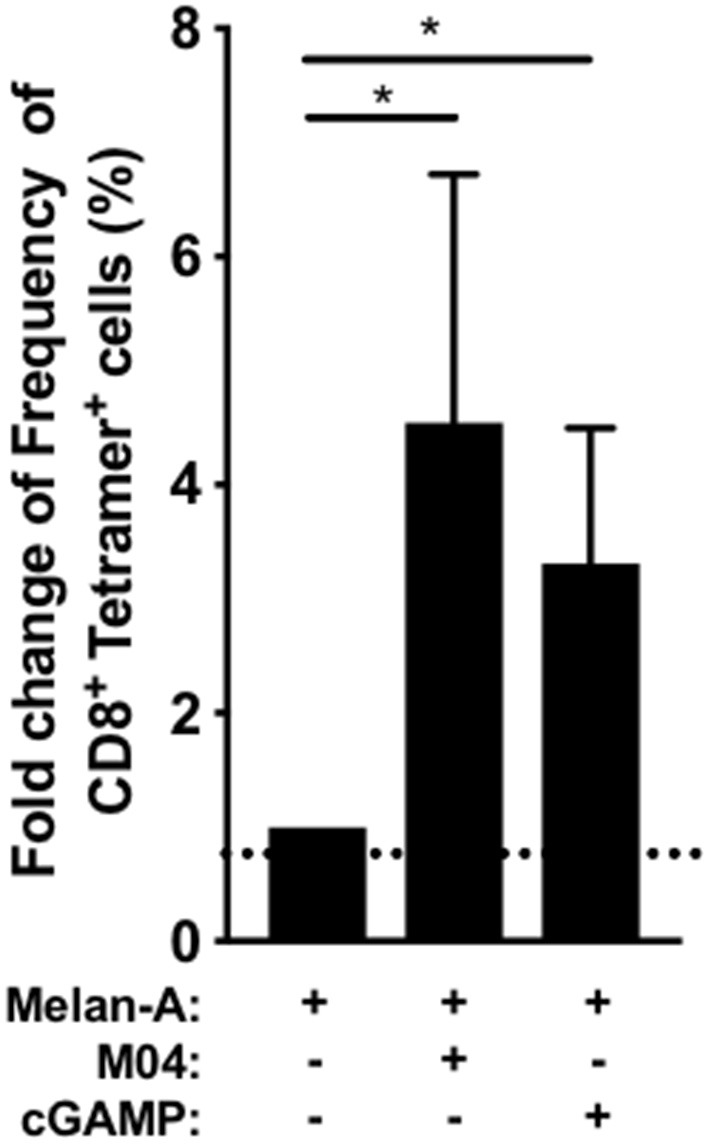
*In vitro* CD8^+^ T cell priming using unfractionated PBMC. Dendritic cell differentiation from healthy HLA-A2+ donor PBMCs (*n* = 7) was induced with GM-CSF (100 ng/ml) and IL-4 (20 ng/ml) and Melan A peptide (1O μg/ml) with M04 (50 μM) or 2,3' cGAMP (5 μg/ml) was added the next day when indicated. On day 11, the primed Metan-A specific CD8^+^ T lymphocytes were detected using tetramer staining within the CD3^+^ T cell population after aggregates and dead cell exclusion. The graph represents the fold increase of Metan-A - specific CD8^+^ T lymphocytes frequency compared to the condition without STING agonists. A non-parametric Friedman signed rank test followed by Dunn's multiple comparison test was used to assess significance. *p < *0.05*.

Potent and adequate CD8^+^ T cell responses against a specific antigen is a key component of the adaptive immune response. Induction of such high-quality T cell responses is crucial for many vaccination objectives. Adjuvants can enhance the function of antigen presenting cells, which through the priming process, will shape the immune response and induce naïve antigen-specific CD8^+^ T cells into potent effector cytotoxic T lymphocytes. In an *ex vivo* assay using cryopreserved and unfractionated PBMCs adapted from (47), we recapitulated the priming, by dendritic cells, of CD8^+^ T cells specific for the model antigen Melan-A, in the presence of M04. Antigen-specific CD8^+^ T cells were detected by Melan-A-tetramer staining. As shown in Figure 11, both M04 and cGAMP induce significantly higher frequencies of primed Ag-specific CD8^+^ T cells compared to the coculture without adjuvant. A 4.5-fold increase was observed in the presence of M04 while cGAMP enhanced this by 3.3 times the frequency of Ag-specific CD8^+^ T cells. These results thus further demonstrate the adjuvant potential of M04 in human primary cells.

### The M04 Transcriptome More Closely Resembles That Induced by cGAMP Than by LPS

**Figure 12:**
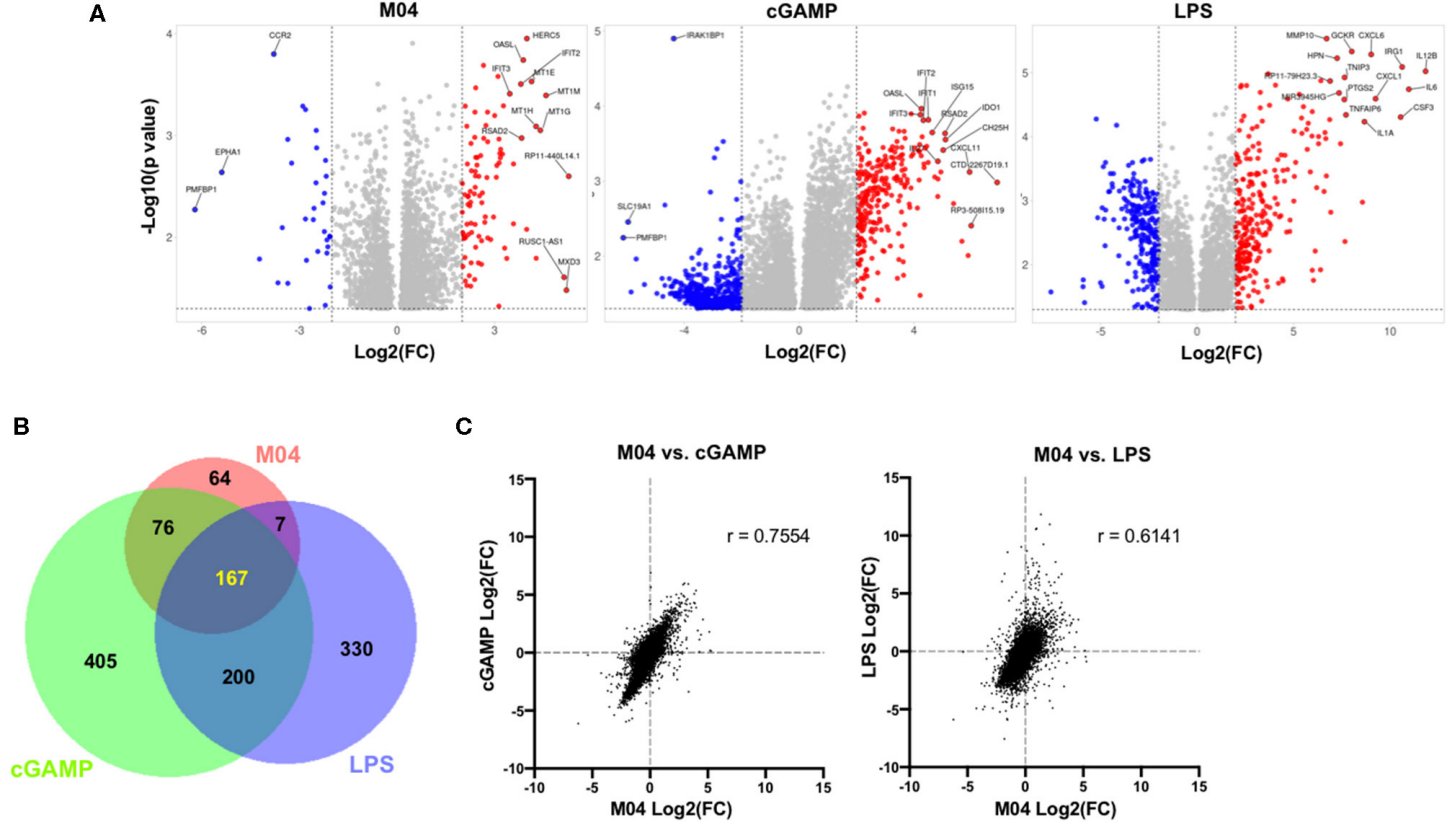
Comparison of transcriptomic changes in PBMCs induced by M04, LPS, and cGAMP. **(A)** Volcano plots illustrating −log_10_(*p* value) and fold change of significantly differentially regulated transcripts for indicated stimulus relative to cells exposed to DMSO vehicle treatment. Gene symbols of the top 15 transcripts as determined by Manhattan distance are labeled; **(B)** Venn diagram illustrating patterns of similarity in indicated stimulus-specific upregulated transcripts; **(C)** Fold change correlation of all detected transcripts between indicated stimuli. Pearson correlation coefficient (r) presented for each comparison.

Given the ability of M04 to induce STING-dependent transcription of targeted ISGs as well as innate phenotypes in primary human cells, we predicted the stimulation of substantial global transcriptional responses by the molecule in PBMCs. We also expected that qualitatively these would more closely resemble those triggered by an agonist of the STING pathway relative to another IRF3-terminal adaptor. To address this, we obtained PBMCs from two healthy human donors and treated them with DMSO vehicle, M04, cGAMP, or the TLR4/TRIF agonist LPS. RNA sequencing was then used to measure individual transcript levels in each sample and comparisons to vehicle-treated cells made (Supplemental Table 1). As shown in Figure 12, M04, cGAMP, and LPS led to the significant more than 2-fold upregulation of 314, 848, and 704 RNA transcripts, respectively. Importantly, however, the number of transcripts induced by M04 that were also uniquely upregulated by the other stimuli was much greater for cGAMP than for LPS (76 vs. 7, respectively). Furthermore, the quantitative similarity in absolute fold changes of all observed transcripts was much higher between M04 and cGAMP (Pearson *r* = 0.7554) than between M04 and LPS (*r* = 0.6141) (Figure 12C). Finally, despite the failure of experiments to show canonical activation of NF-κB by M04 in fibroblasts, treatment of PBMCs with the compound led to induction of multiple RNAs whose transcription was predicted by two different computational tools (PASTAA and RegulatorTrail) to be associated with activity of this transcription factor (Supplemental Tables 2 and 3) (48, 49). This is consistent with previous work describing NF-κB activation by STING (8, 50, 51). We hypothesize that our results likely represent a phenomenon related to intrinsic differences in cell type whereby stromal and non-stromal cells are differentially reactive to NF-κB-associated, STING-mediated pro-inflammatory responses. This will require follow up mechanistic efforts to dissect, however.

## Discussion

The innate and adaptive immunostimulatory potential of synthetic STING activation has greatly incentivized the discovery and characterization of novel molecular entities that stimulate this pathway for anti-cancer therapies (45, 52) and as a strategy to enhance vaccination (53, 54). Currently the most clinically well-developed STING inducers are dithio-mixed linkage derivatives of cyclic dinucleotides such as ML-RR-S2 CDA (also known as ADU-S100) that are in clinical trials (NCT03172936) (55). Unfortunately, CDNs exhibit chemical liabilities including violation of Lipinski rules (56) for druglikeness, susceptibility to phosphodiesterase-mediated degradation (57, 58), and their size, hydrophilicity, and negative charge render them impermeable to cell membranes thus impairing exposure to cytosolic STING (59, 60). In general, the properties of small molecules such as M04 mitigate these issues and, as such, may ultimately represent a superior strategy for activating STING-mediated processes *in vivo*.

Our work demonstrates that M04 activates an innate response in human cells that requires STING and IRF3 but not an array of other described cytosolic PRRs of DNA (in particular cGAS). M04 also does not induce synthesis of cGAMP by a cGAS-dependent or independent process. Moreover, in addition to the loss of function approach used to demonstrate protein essentiality, we also used forward genetics methods that demonstrated conference of M04 responsiveness to non-responsive cells (including mouse cells) following ectopic expression of hSTING. These results strongly argue that the compound's mechanism of action involves direct engagement of the STING protein. Why thermal shift analysis showed no M04-mediated enhancement of STING-CTD stability is not clear but it is possible that the compound binds to a protein domain outside this region that leads to activation.

We also show that M04 is capable of inducing innate activation in a manner that requires specific variants of human STING. Surprisingly, diABZI showed similar patterns of allele-specific efficacy with poor responsiveness in STING-HAQ and normal activity in STING-R232H and STING-WT. This result is actually inconsistent with what was previously shown for diABZI in primary human cells homozygous for these alleles (26). Whether this disparity is associated with the cell type or model system we employed will require additional follow up studies. Overall, however, these data suggest that it is possible to identify small molecules that exhibit allele-specific activity, which may be important given the existing STING polymorphism in the human population (39). Interestingly, the alleles with which M04 and diABZI function best encode arginine or histidine amino acids at position 232 and arginine at position 293, which are both within the LBD. STING-HAQ differs from these alleles by encoding glutamine at position 293, alanine at 230 and histidine at position 71, which is in the transmembrane domain. Whether any of these individually are associated with our observations regarding M04 or diABZI activity will require additional examination. Furthermore, there exist other naturally occurring variants that exist with relatively high frequency that we also plan to examine. Surprisingly absent from the archive of studies examining the clinical value of STING agonists is exploration of genetic impacts. Given the breadth and frequency of human polymorphism in this protein as well as links between phenotype and variant, a more penetrative consideration of how this will affect STING-based therapeutics is clearly warranted. Understanding the spectrum of allele-associated molecule reactivity will be a very important step in the development of STING-directed pharmacophores.

It is worth noting that while STING activation is known to facilitate establishment of an adaptive immune response, its overall immune impact is more nuanced. In some models the protein is associated with tolerogenic responses likely through its induction of indoleamine 2,3-dioxygenase, as observed in our transcriptomic data for both M04 and cGAMP (61, 62). However, determining with precision the immune-mediated effects conferred by M04 and whether they have potential clinical utility is hindered by its inactivity in mice. Fortunately, primary human cells do display responses to M04 such as cytokine secretion, expression of DC maturation markers, and T cell cross-priming that associate with conventional immune activity. Previous work has identified similar phenomena induced by CDN-based agonists (63–65) as well as dsDNA (66) and diABZI (26). Why some of the DC markers were not induced as expected is unclear and could be related to donor-specific effects such as STING genotype or a skewed set of molecular processes induced in primary tissues by the compound. The key question regarding M04 in this regard is whether the innate processes induced by the molecule *ex vivo* would translate into meaningful immunological effects *in vivo*. If M04 elicits activity by binding directly to STING as we predict, obtaining this answer may be possible in mice that express hSTING. Previous work has shown that hSTING is functional and responsive to activating ligands in mouse cells (44). It is therefore possible that replacement of the endogenous mouse STING with a human allele could lead to an animal model useful for characterizing molecules with human but not mouse specificity (17, 19, 67). Accordingly, this would greatly expand the spectrum of potential compounds that could be explored for therapeutic activity.

## Materials and Methods

### Reagents and Antibodies

Dimethyl sulfide (DMSO) was purchased from Thermo Fisher. Human recombinant IFNβ was obtained from PBL. Lipopolysaccharide (LPS) was obtained from Sigma. cGAMP was obtained from Invivogen. Stocks of M04 were originally obtained from Enamine. Larger stocks of M04 and ABZI were synthesized by the OHSU Medicinal Chemistry Core Facility. diABZI was obtained from MedChem Express. Puromycin was obtained from Invivogen and used at 3 μg/mL in resistant cell culture. Steady-Glo cell lysis/luciferin and CellTiter-Glo viability assay kits were obtained from Promega. Lipofectamine 3000 was obtained from Invitrogen. Sources and concentrations of antibodies used against the following antigens are indicated in parentheses: glyceraldehyde-3-phosphate dehydrogenase (GAPDH) (SC-51906; Santa Cruz) (1:10,000), IRF3 (4302; Cell Signaling), human phospho-IRF3 (76493; Abcam), mouse IRF3 (SC-9082; Santa Cruz), mouse phospho-S379 IRF3 (79945S; Cell Signaling), mouse phospho-S396 IRF3 (29047S; Cell Signaling), STING (13647S; Cell Signaling), phospho-S366 STING (19781S; Cell Signaling), TBK1 (3504S; Cell Signaling), phospho-TBK1 (5483S; Cell Signaling), NF-kB P65 (SC372; Santa Cruz), NF-kB P50 (3035; Cell Signaling), GM-130 (610823; BD Biosciences).

### Cell Line Cultures and Virus

Telomerase-transduced human foreskin fibroblasts stably transduced with the IFN-responsive pGreenFire-ISRE lentivector (System Biosciences) were used as previously described (18, 19). A549 and HEK293T cells were a gift from Jay Nelson (Oregon Health and Science University). MonoMac6 (MM6) cells were a kind gift from Michael Gale (University of Washington) and used as described (17). THP-1-ISG-Lucia and RAW-ISG-Lucia were obtained from Invivogen. HEK293T, A549, THF, and RAW264.7 cells were maintained in Dulbecco's modified Eagle's medium (DMEM) containing 10% fetal bovine serum (FBS), penicillin (100 U/ml), and streptomycin (100 U/ml) and were transduced with a lentivector containing the pGreenFire ISRE cassette. THP-1 and MM6 cells were maintained in RPMI 1640 medium supplemented with 10% FBS, penicillin (100 U/ml), streptomycin (100 U/ml), and HEPES (10 mM). THP-1 ISG-lucia cells were differentiated by 2 h of treatment with 100 ng/mL PMA, and then the PMA was removed and replaced with complete medium for 72 h of incubation prior to all assays. All cells were grown at 37°C and 5% CO_2_. Sendai virus (SeV) was obtained from Charles River Laboratories and used at 160 hemagglutination units (HAU)/ml. Human cytomegalovirus was grown and titered as described (68) and exposed to cells at a multiplicity of infection (MOI) of 3 unless otherwise indicated. cGAMP was transfected into cells using Lipofectamine 3000 following the manufacturer's protocol.

### CRISPR/Cas9-Mediated Genome Editing and Ectopic Gene Expression

Genome editing using lentivector-mediated delivery of CRISPR/Cas9 components was performed as described previously (17–19). Briefly, we used the lentiCRISPRv2 vector (a gift from Feng Zhang; Addgene plasmid # 52961) (69). STING-specific guide RNAs (gRNA) were cloned into this vector (mouse STING gRNA: AGTATGACCAGGCCAGCCCG; human STING gRNA: CCCGTGTCCCAGGGGTCACG) and was then used to transduce the appropriate cells, selected using puromycin, and knockout validated by immunoblot. Stable and transient expression of hSTING variants was done by cloning target ORFs into the pLVX-EIF1α vector. Cells were then transduced and selected for antibiotic resistance as described previously (70). Transient transfection of these vectors into HEK293T cells was done using Lipfectamine 3000 according to the manufacturer's protocol (Invitrogen).

### Luciferase Reporter Assay and Type I Interferon Bioassays

For reporter assays cells (THF-ISRE, RAW264.7-ISG-Lucia, THP-1-ISG-Lucia) were plated in white 96-well plates 24 h before stimulation. Treatments were performed in quadruplicate in 50 μL of either DMEM or RPMI plus 2% FBS overnight unless otherwise indicated. Steady-Glo lysis/luciferin reagent (Promega) was added (1:1 [vol/vol]) to each well, and luminescence was measured on a Synergy plate reader (BioTek). For cell viability assays, CellTiter-Glo reagent was used following the manufacturer's suggested protocol. For type I IFN bioassays, cells of interest were plated at 50,000 cells/well in 24-well-plates and serum starved in X-Vivo15 medium for 1 h prior to treatment. After treatment for 24 h, the media was harvested and clarified at 10,000 x g for 3 min. Recombinant IFNβ (at 40, 20, 10, 5, 2.5, 1.25, and 0.63 U/ml) was used to generate a standard response curve. The supernatant or standard was then added to THF-ISRE-ΔIRF3 cells (do not respond to STING/IRF3-inducing stimuli) plated as described above for 8 h, and luminescence was measured. IFN was quantitated by curve fitting relative to the signals generated from the standards.

### Immunoblotting

Sodium dodecyl sulfate-polyacrylamide gel electrophoresis (SDS-PAGE) and immunoblotting were performed as follows. After cell pelleting at 2,000 × g for 10 min, whole-cell lysates were harvested in RIPA lysis buffer (50 mM Tris-HCl [pH 8.0], 150 mM NaCl, 1% NP-40, 0.5% sodium deoxycholate, and 0.1% SDS) supplemented with Halt protease and phosphatase inhibitor cocktail (Thermo Fisher). Lysates were electrophoresed in 8% polyacrylamide gels and then transferred onto polyvinylidene difluoride (PVDF) membranes (Millipore) by semidry transfer at 15 V mA for 15 min. The blots were blocked at room temperature for 2 h or overnight, using 5% non-fat milk in PBS containing 0.1% Tween 20. The blots were exposed to primary antibody in 5% non-fat milk in PBS containing 0.1% Tween 20 for 18 h at 4°C. The blots were then washed in PBS containing 0.1% Tween 20 for 20, 15, and 5 min, followed by deionized water for 5 min. A 1-h exposure to horseradish peroxidase-conjugated secondary antibodies and subsequent washes were performed as described for the primary antibodies. Antibodies were visualized using enhanced chemiluminescence (Pierce).

### Indirect Immunofluorescence Assay

For the indirect immunofluorescence assays (IFA), cells were grown on coverslips in 24-well-plates and treated as described above. At room temperature, cells were washed twice with PBS, fixed for 30 min in 3.7% formalin, washed, and quenched for 10 min using 50 mM NH_4_Cl. Cells were permeabilized with 0.1% Triton X-100 for 7 min and washed three times with PBS containing 2% bovine serum albumin (BSA). Cells were incubated with primary antibody in PBS containing 2% BSA at 37°C for 1 h, washed three times in PBS containing 2% BSA (10 min for each wash), and incubated with fluorescently conjugated secondary antibody diluted 1:1,000 in PBS containing 2% BSA for 1 h. Cells were washed twice in PBS containing 2% BSA (10 min for each wash) and once in PBS. Coverslips were mounted on a microscope slide with Vectashield mounting medium (Vector Laboratories, Burlingame, CA) containing DAPI, and imaging was performed on an Evos cell-imaging system.

### RNA Isolation and Semiquantitative Reverse Transcription-PCR

Total RNA was isolated from cells, treated with the DNase provided in a DNA-free RNA isolation kit (Zymo Research) according to the manufacturer's protocol, and quantified by UV spectrometry. Single-stranded cDNA for use as a PCR template was made from total RNA and random hexamers to prime first-strand synthesis via a RevertAid First Strand cDNA synthesis kit (Thermo Fisher). Comparison of mRNA expression levels between samples was performed using semiquantitative real-time reverse transcription-PCR (qPCR) with an Applied Biosystems sequence detection system according to the ΔΔC_T_ method (71), with GAPDH as a control. Prevalidated Prime-Time 6-carboxyfluorescein qPCR primer/probe sets obtained from IDT were used for all genes.

### STING Protein Purification and Thermal Shift Assays

Assays of the molecular interaction between the purified human STING C-terminal domain (amino acids 137 to 379; non-transmembrane domain) and M04 were performed as previously reported (19). Briefly, the 6xHis STING CTD open reading frame was cloned into pRSET-B (Invitrogen) and used to transform the Escherichia coli strain pLysS (Promega). The transformed *E. coli* cells were then induced to express the protein as induced by 1 mM IPTG (isopropyl-D-thiogalactopyranoside) at 16°C for 18 h. STING protein was purified by nickel-affinity chromatography (Clontech Laboratories) and then further purified by gel filtration chromatography (HiPrep 16/60 Sephacryl S-100 HR column; GE Healthcare Life Sciences). Eluted proteins were concentrated using Amicon centrifugal filters (10-kDa cutoff). For thermal shift assays, SYPRO Orange dye was used, following the manufacturer's suggested protocol, to determine protein stability in the presence and absence of cGAMP (Invivogen) or M04.

### Human Samples

Peripheral blood mononuclear cells (PBMCs) were collected and analyzed at two separate institutions for cytokine secretion measurements, maturation marker analysis, and cross-priming assays. All procedures were performed accordance with the Institutional Review Boards of the respective institutions (Drexel University College of Medicine, Earle A. Chiles Research Institute). Donor samples analyzed at Drexel University were obtained from Martin Health System (Stuart, Florida). The study was approved by the Institutional Review Board of Martin Health System and Drexel University College of Medicine (Philadelphia). All donors signed informed consent from all participants. Patients with locally advanced or borderline resectable pancreatic cancer enrolled on a clinical study (46) provided a pre-treatment blood sample. Studies were approved by the institutional review board at Providence Portland Medical Center, Portland OR with study ID numbers PHS 10-141B and PHS 13-026A. The clinical trial registration numbers are NCT01342224 and NCT01903083. All patients provided written informed consent for treatment and participation in these studies, including analysis of serum and blood parameters over the course of the study.

### Luminex Analysis

PBMCs were plated at 4 × 10^5^ per well in 96-well-plates, stimulated with DMSO, M04 (50 μM), LPS (100 ng/mL), or cyclic-di-AMP (10 μg/mL) diluted in RPMI, and incubated at 37°C and 5% CO_2_ for 24 h. Supernatants were then removed and used in a multiplex cytokine bead-based assay according to the manufacturer's protocol (BD Biosciences human inflammation cytokine bead array, catalog number 551,811, or BioLegend human IL-12p70 enzyme-linked immunosorbent assay [ELISA] Max).

### Generation of Human Monocyte-Derived Dendritic Cells (mDCs)

Human PBMCs from healthy donors were obtained immediately after blood withdrawal using the Ficoll-Paque (GE Healthcare) gradient method and stored in liquid nitrogen until usage. Cells were thawed in RPMI 1640 (Corning) supplemented with 10% fetal bovine serum (Access Biologicals) and 1% penicillin/streptomycin (Gibco). CD14^+^ CD16^+^ monocytes were then enriched from total PBMCs by negative selection using the EasySep^TM^ human monocyte enrichment kit without CD16 depletion (STEMCELL Technologies) according to the manufacturer's protocol and counted. Cells were then resuspended in serum-free CellGenix GMP dendritic cell medium (CellGenix) with 100 ng/ml of recombinant human GM-CSF (BioLegend) and 20 ng/ml of recombinant human IL-4 (Gemini Bio-Products) at a density of 2 × 10^6^ per ml in 24-well plates. After 48 h of incubation, cells were stimulated with 0.5 ug/ml of LPS (Invivogen) plus 40 ng/ml of IFNγ (Gemini Bio-Products) in medium or with two concentrations 25 and 50uM of the STING agonist MO4 (source) in DMSO, and compared to the DMSO control. Dendritic cells were harvested after 24 h of stimulation, and analyzed by flow cytometry.

### Flow Cytometric Analysis of Stimulated Human mDCs

Harvested mDCs were incubated with TruStain FcγR block (BioLegend) and fluorochrome-conjugated antibodies for 15–20 min on ice in the dark. The following BioLegend fluorochrome-conjugated anti-human antibodies were used: CD3 (clone HIT3α), CD19 (clone HIB19), CD14 (clone M5E2), CD11c (clone 3.9), HLA-DR (clone L243), CD86 (clone IT2.2), CD83 (clone HB15e), CD40 (clone 5C3), and CD80 (clone 2D10). Dead cells were identified using both LIVE/DEAD fixable Aqua Dead Cell Stain Kit for flow cytometry (Vivid) (Life Technologies) and Annexin V (BD Biosciences). mDC samples were washed then resuspended in PBS plus 2% FBS then acquired on a BD LSR II and analyzed with FlowJo software (Treestar). The gating strategy excluded doublet cells and mDCs were gated on live (Vivid^−^ Annexin V^−^) CD3^−^ CD19^−^ CD11c^+^ cells.

### Peptides

The HLA-A2-restricted Melan-A/ MART-1 modified peptide (ELAGIGILTV, residues 26-35_A27L_) was used for *in vitro* priming and was obtained from Biosynthesis. The tetramer HLA-A^*^02:01- ELAGIGILTV (Melan-A/MART-1) was obtained from the NIH Tetramer Core Facility (Emory University).

### *In vitro* Priming of Naive Melan-A/MART-1 Ag-Specific CD8^+^ T Cells

Naïve CD8^+^ T cells precursors for the Melan-A/MART-1 epitope ELA were primed *in vitro* using unfractionated PBMC protocol as described in [1] with minor modifications. Briefly, PBMC from HLA-A2^+^ healthy donors were thawed and seeded at 5 × 10^6^ cells/ml in CellGro® DC medium (CellGenix™), supplemented with human GM-CSF (100 ng/ml; MACS Miltenyi Biotec) and IL-4 (20 ng/ml; Gemini Bio-products) in a 24-well tissue culture plate. After 24 h, Melan-A/MART-1 antigen (at 10 μg/ml) was added, in presence of M04 (final 50 μM) or 2'3'cGamp (5 μg/ml, Invitrogen) to induce maturation of resident dendritic cells. Twenty-four hours later and every 3 days, half of the media was replaced by fresh RPMI 1640 (Corning) supplemented with 8% human serum (Atlanta Biologicals) and IL-2 (20 U/ml, Miltenyi Biotech). On day 11, the CD8^+^ T cells frequency and were assessed by flow cytometry within the CD3^+^CD8^+^ T cell population using Melan-A –HLA-A2 tetramer staining after exclusion of dead cells.

### *In vivo* Administration of M04

All animal procedures for *in vivo* administration of M04 were conducted in accordance with and approved by the Oregon Health and Science University Institutional Animal Care and Use Committee (IACUC) under protocol 0913. The Oregon Health and Science University IACUC adheres to the NIH Office of Laboratory Animal Welfare standards (OLAW welfare assurance A3304-1). C57BL/6J mice (5–7 weeks of age; Jackson Laboratories) were housed in cage units, fed *ad-libitum*, and cared for under USDA guidelines for laboratory animals. M04 at 25 mg/kg of body weight or DMXAA (or DMSO alone) was prepared in DMSO plus PBS to 200 μL and injected intraperitoneally. Animals were euthanized at 5 h post-injection by isoflurane overdose. Spleens were harvested, RNA isolated, and qPCR performed as described above.

### RNA-seq

PBMCs from two healthy adult donors were obtained from StemCell Technologies, grown in 12 well-dishes in RPMI + 10% FBS, and treated in duplicate for 6 h with 1% DMSO vehicle, 50 μM M04, 100 ng/mL LPS, or 15 μg/mL cGAMP. Total RNA was isolated using Direct-zol RNA mini-prep kit (Zymo Research) in accordance with manufacturer's instructions and profiled for intactness on a Bioanalyzer (Agilent). Libraries were then prepared using the Tru-Seq RNA Sample Preparation kit (Illumina). Briefly, poly(A)+ RNA was isolated from 500 ng of total RNA per sample. The isolated RNA was fragmented using divalent cations and heat. First strand cDNA was generated using random hexamer priming. The RNA template was removed and the second strand was synthesized. The ends of the cDNAs were repaired, followed by adenylation of the 3′ termini. Indexing adapters were ligated to the cDNA ends. The ligation products were amplified using polymerase chain reaction (PCR). The amplification product was cleaned using AMPure XP beads (Beckman Coulter). Libraries were profiled on the Tapestation 2200 (Agilent). The concentration of the libraries was determined using real time PCR on a StepOne or StepOnePlus Real Time PCR Workstation (Thermo) using a library quantification kit (Kapa Biosystems). Samples were mixed for multiplexing and run on a HiSeq 2500 (Illumina) using a 100 cycle single read protocol. Base call files were converted to fastq format using Bcl2fastq (Illumina).

### Gene Expression and Transcription Factor Prediction Analysis

The quality of the raw sequencing files were first evaluated using FastQC combined with MultiQC (72) (http://multiqc.info/). The files were imported into the Oregon National Primate Center's DISCVR-Seq, LabKey (73) server-based system, PRIMe-Seq. Trimmomatic (74) was used to remove any remaining Illumina adapters. Reads were aligned to the Homo_sapiens.GRCh38 genome in Ensembl along with its corresponding annotation, release 84. The program STAR (v020201) (75) was used to align the reads to the genome. STAR has been shown to perform well-compared to other RNA-seq aligners (76). Two-pass mode was used with default parameters. Since STAR utilizes the gene annotation file, it calculated the number of reads aligned to each gene. RNA-SeQC (v1.1.8.1) (77) was utilized to ensure alignments were of sufficient quality. Samples had an average of 45 M mapped reads, an average exonic rate of 83%, and an average of 22 K genes detected (>5 reads) per sample. Gene-level raw counts were normalized using DEseq2 (78) which were then transformed using regularized log transformation (rlog) to stabilize variance in R. After data processing, gene-wise general linear models with compound symmetry covariance structure was used (to account for repeated response measures on the same subject) to identify differentially expressed genes in SAS9.4. We used criteria to designate genes as differentially regulated in each stimulus vs. control with fold change ≥ 2 (up or down) and raw *p* < 0.05. Transcription factor prediction analysis was performed by submitting all genes found to be significantly upregulated by M04 to the online tools PASTAA and RegulatorTail (48, 49) using default settings. Venn diagrams were made using BioVenn (79). Volcano plots were made using VolcanoNoseR (https://huygens.science.uva.nl/VolcaNoseR/).

## Data Availability Statement

The raw data supporting the conclusions of this article will be made available by the authors, without undue reservation. The transcriptomics data has been deposited in the Gene Expression Omnibs under accession Series (GSE152179).

## Ethics Statement

The animal study was reviewed and approved by Oregon Health and Science University IACUC.

## Author Contributions

VD, MG, LT, AN, JB, JA, and BG: conceived of experiments. JA, SB, NM, KP, BG, DB, TS, HJ, JB, MC, and CS: performed experiments. JA, AN, MG, LT, EH, and VD: interpreted experiments. VD: wrote manuscript. All authors contributed to the article and approved the submitted version.

## Conflict of Interest

The authors declare that the research was conducted in the absence of any commercial or financial relationships that could be construed as a potential conflict of interest.
