# Supplementary figures and images for: Characterization of a Novel Compound That Stimulates STING-Mediated Innate Immune Activity in an Allele-Specific Manner
Source: Front Immunol. 2020 Jul 8;11:1430. doi: 10.3389/fimmu.2020.01430 (PMC7360819; doi:10.3389/fimmu.2020.01430)

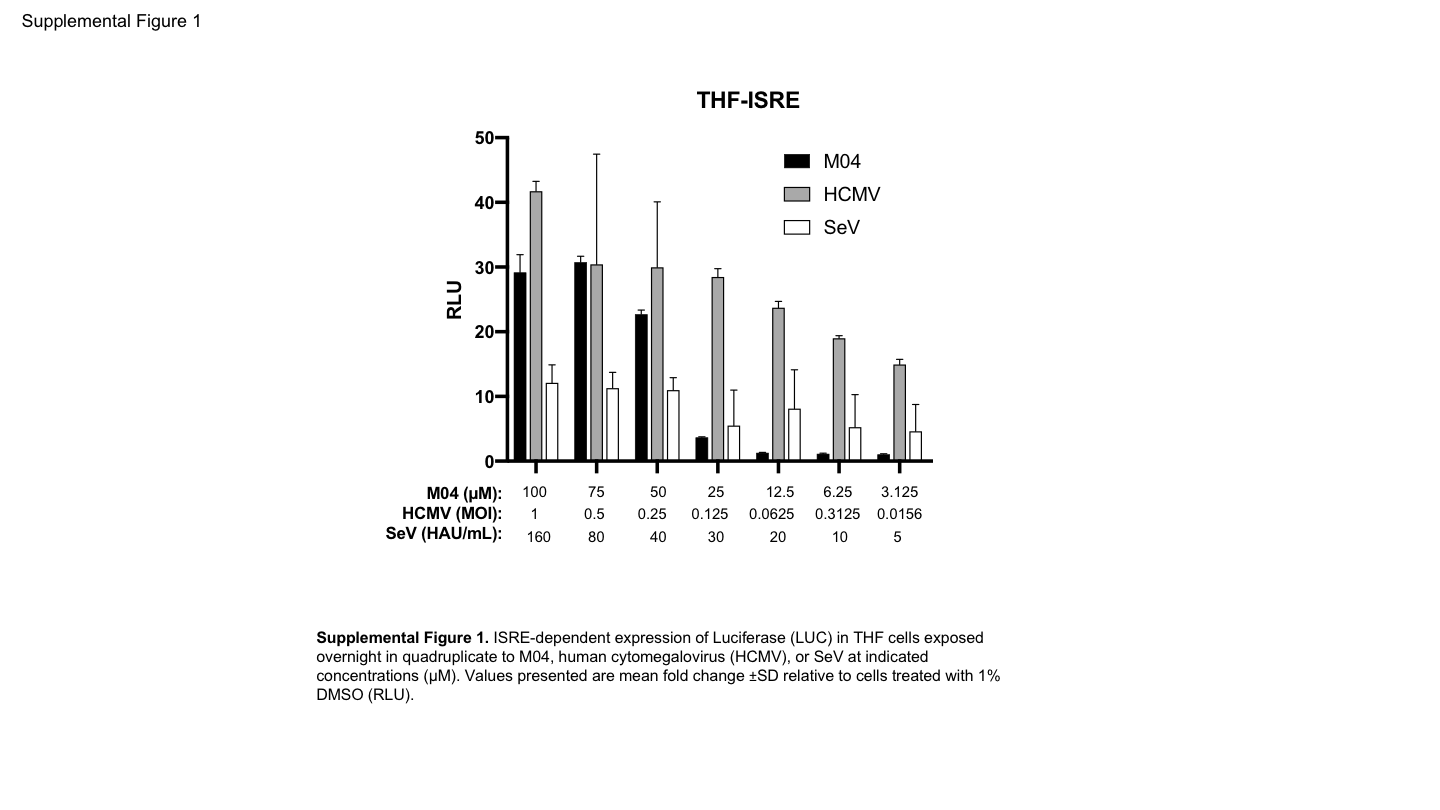

Supplement: Supplementary file 1 [file Image_1.TIFF]

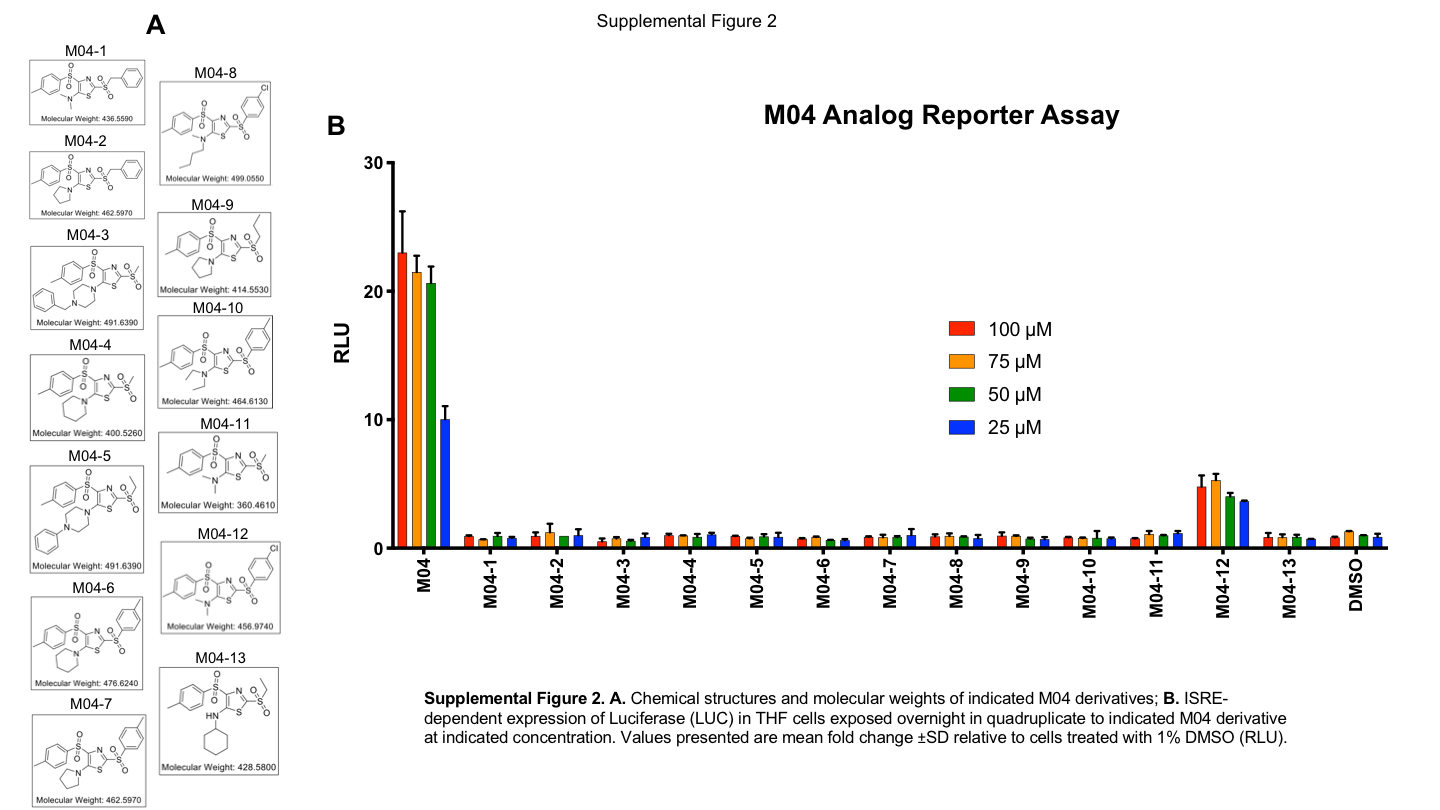

Supplement: Supplementary file 2 [file Image_2.TIFF]
